# Exploring Functionally Enhanced BLP‐Trained Macrophage Subpopulations in *S. Aureus* Infection: Underlying Mechanisms and Therapeutic Significance

**DOI:** 10.1002/advs.202417142

**Published:** 2025-10-21

**Authors:** Yantong Wan, Yinghao Hong, Xiangjun Ji, Jing Xiang, Jinxi Liu, Lixin Liang, Meng Ren, Wenhan Chen, Tengfei Xu, Zhijie Li, Tieliu Shi, Yong Jiang, Huaping Liang, Jinghua Liu

**Affiliations:** ^1^ Guangdong Provincial Key Laboratory of Proteomics, Department of Pathophysiology, School of Basic Medical Sciences Southern Medical University Guangzhou Guangdong 510515 China; ^2^ State Key Laboratory of Trauma and Chemical Poisoning Daping Hospital Army Medical University Chongqing 400038 China; ^3^ Center for Bioinformatics and Computational Biology The Institute of Biomedical Sciences and School of Life Sciences East China Normal University Shanghai 200062 China; ^4^ Department of Geriatric Medicine Shenzhen People's Hospital The Second Clinical Medical College Jinan University Shenzhen Guangdong 518020 China; ^5^ Department of Respiratory and Critical Care Medicine The Tenth Affiliated Hospital Southern Medical University Dongguan Guangdong 523018 China

**Keywords:** antioxidative stress, BLP, macrophage, NRF2, single‐cell transcriptomics, trained immunity

## Abstract

Tolerance to bacterial lipoprotein (BLP) is an evolved protective mechanism characterized by an enhanced resistance of BLP‐trained macrophages to microbial infection. However, the underlying mechanisms are not fully understood, and their potential for translational clinical application needs further evaluation. In the present study, through single‐cell RNA sequencing (scRNA‐seq), transcriptomic profiles in both naïve and BLP‐trained bone marrow‐derived macrophages (BMDMs) during *Staphylococcus aureus* infection are analyzed, and 13 distinct BMDM subpopulations are identified. Notably, BLP‐trained tolerance initiates the emergence of two novel BMDM subpopulations, C5 and C7, characterized by increased antibacterial gene expression and enhanced anti‐inflammatory and antioxidative stress abilities. Moreover, BLP‐trained BMDMs demonstrate activation of the NRF2 signaling pathway, thereby augmenting an antioxidative stress response and mitigating oxidative stress‐induced cell damage and ferroptosis, while undergoing metabolic reprogramming characterized by enhanced glycolysis and oxidative phosphorylation pathways, together with increased anti‐inflammatory metabolites. Critically, in vivo adoptive transfer of BLP‐trained BMDMs protects mice against sepsis‐associated lethality by attenuating systemic inflammatory response, accelerating bacterial clearance, and alleviating organ damage. Collectively, the present study presents a single‐cell atlas of murine BMDMs at rest and under *S. aureus* infection following BLP training, which reveals novel mechanisms of BLP training‐altered macrophage immunity and identifies macrophage subpopulations responsible for an enhanced resistance to infection, thus offering new preventive and therapeutic strategies for sepsis.

## Graphical abstract

BLP‐trained macrophages show augmented resistance to infection, but their mechanisms remain unclear. scRNA‐seq identified 13 BMDM subsets, with two novel anti‐inflammatory/antibacterial subpopulations (C5/C7) emerging post‐BLP training. BLP training activates NRF2, attenuates oxidative stress/ferroptosis, and boosts glycolysis/OXPHOS. Adoptive transfer of BLP‐trained BMDMs protects mice against sepsis‐associated lethality by limiting inflammation/organ damage, revealing therapeutic potential.

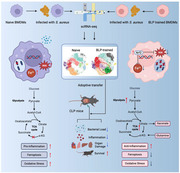

## 1 Introduction

The pathological mechanism(s) of sepsis are complex, often culminating in high mortality rates, and currently, there are no clinically approved drugs that specifically target this disease.^[^ ^1^ ^]^ Our previous work has shown that pretreatment or “training” of innate immunity with bacterial lipoprotein (BLP), a Toll‐like receptor 2 (TLR2) agonist, confers robust protection against sepsis‐associated lethality both in bacterial infection‐induced sepsis and cecal ligation and puncture (CLP)‐induced polymicrobial sepsis.^[^ ^2^ ^]^ This protection afforded by BLP pretreatment or training is closely associated with the enhanced phagocytic and bactericidal capabilities of innate phagocytes such as monocytes/macrophages in vitro^[^ ^3^, ^4^ ^]^ and attenuated systemic inflammatory responses and accelerated bacterial clearance from the circulation and visceral organs in vivo.^[^ ^5^ ^]^ However, all these results and the derived conclusions are principally based on the premise that monocytes/macrophages are a homogeneous cell population in nature, and their immune response status, either decreased vs increased or suppressed vs activated, is simply determined by the average or mean immune response of each cell in one cell population that is assumed to be the same homogeneous. Nevertheless, this assumption fundamentally ignores the inherent heterogeneity of so‐called one‐cell populations with the same cell surface markers, especially the heterogeneity of the immune cell population. Therefore, there is an urgent need for new technologies and methods to distinguish various macrophage phenotypes, identify their heterogeneity, and help us better understand the complex process of how macrophages respond to bacterial infection and protect against the invasion of various pathogenic microorganisms.

Single‐cell RNA sequencing (scRNA‐seq) has emerged as a pivotal technology to sequence the transcriptome of a single cell and obtain a large amount of cellular transcriptomic information at the same time. Since scRNA‐seq allows us to see the full picture of the genes expressed at the individual cell level, thereby enables us to further discover and reveal the heterogeneity of the innate immunocyte population.^[^ ^6^ ^]^ Based on single‐cell transcriptomics, researchers are now unraveling the origin and diversity within macrophage populations.^[^ ^7^ ^]^ Two major subtypes of macrophages are prevalent across almost all tissues. The first is the tissue‐resident macrophages, which originate from embryonic precursors and persist in tissues through self‐renewal. The other type is the monocyte‐derived macrophages, which are differentiated from bone marrow‐derived monocytes after they migrate into the infected or injured tissue upon tissue injury and infection.^[^ ^8^ ^]^ Bone marrow‐derived macrophages (BMDMs) are one of the most commonly used primary macrophages in the laboratory. Currently, it is unclear whether these BMDMs display different cell subtypes or phenotypes in the resting state, whether the subtypes or phenotypes of BMDMs change upon bacterial infection, and which cell subtype or phenotype is involved in and responsible for the immune response to bacterial infection. More importantly, it is unclear whether BLP training affects the distribution of macrophage subtypes and induces new phenotypes, especially the relationship between the altered subtypes or phenotypes and the previously observed enhanced bactericidal ability in BLP‐trained BMDMs. In the present study, we employed scRNA‐seq for transcriptomic analysis of BLP‐pretreated (BLP‐trained) vs untreated (naive) murine BMDMs in the resting status and upon *Staphylococcus aureus* (*S. aureus*) infection for various time periods. Consequently, we obtained 13 distinct BMDM subpopulations with a series of statuses at rest and during activation, highlighting the complexity beyond the traditional M1 and M2 polarization states. Significantly, we discovered that BLP training induced the emergence of two novel subpopulations, C5 and C7, characterized by substantially increased expression of anti‐inflammatory and antibacterial genes, augmented antioxidative stress ability, and enhanced chemokine transcription. Notably, we observed that BLP‐trained macrophages exhibited strong activation of the nuclear factor erythroid 2–related factor 2 (NRF2) signaling pathway, thereby augmenting the antioxidative response and attenuating oxidative stress‐associated cell damage and ferroptosis. Interestingly, we found that BLP‐trained macrophages underwent a shift of intracellular metabolic pathways with significantly enhanced both glycolysis and tricarboxylic acid (TCA) cycle, accompanied by increased anti‐inflammatory metabolites itaconic acid and its derivative citric acid. Remarkably, adoptive transfer of BLP‐trained macrophages conferred protection against sepsis‐associated lethality, with reduced inflammatory cytokine release, diminished bacterial burden in the circulation and visceral organs, and attenuated tissue and organ injury. These findings provide a new perspective for our understanding of the macrophage heterogeneity as well as phenotypic characteristics and functional changes of macrophage subpopulations in immune response to pathogenic bacterial infection, which are pivotal for further revealing the mechanism(s) underlying BLP training‐afforded protection and will offer new ideas and approaches in immune modulation therapies for microbial infection and sepsis.

## 2 Results

### 2.1 scRNA‐seq Reveals Macrophage Heterogeneity Upon *S. Aureus* Infection and Following BLP Training

**Figure 1:**
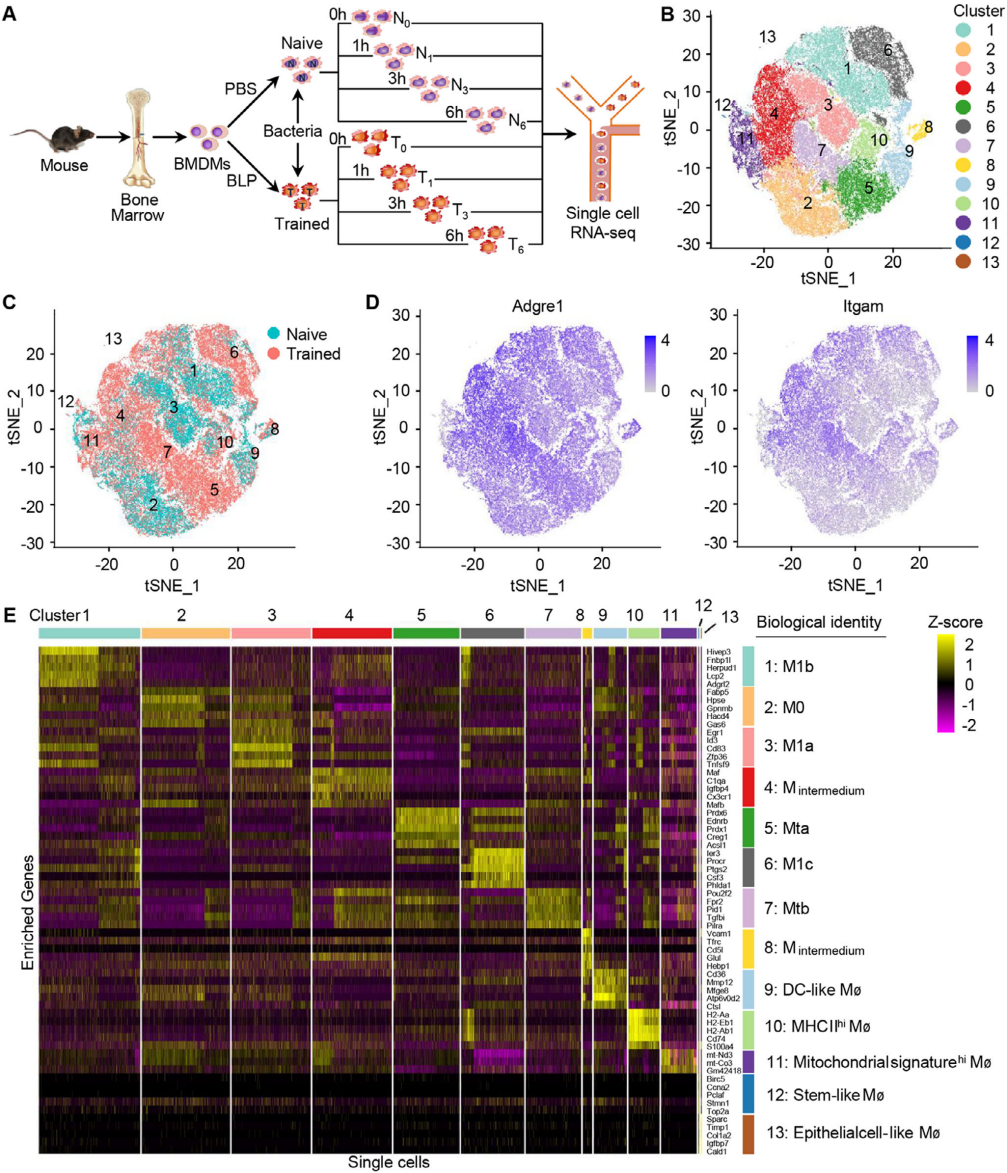
Single‐cell transcriptional profiling identifies 13 populations across naive and BLP‐trained macrophages. A) Experimental process for single‐cell transcriptome sequencing. B) T‐distributed stochastic neighbor embedding (tSNE) plot shows clustering of 79596 macrophages based on gene expression. Point coordinates are based on tSNE dimensionality reduction of the top 15 principal components. C) Naïve and BLP‐trained macrophages overlaid on the tSNE plot. D) Normalized expression of macrophage markers (Adgre1, Itgam) overlaid on tSNE plot. E) Cell type signatures are shown in the heatmap in the relative expression level. The color scale of genes (rows) across cell clusters (columns) is shown.

In the present study, scRNA‐seq of naive (N) and BLP‐trained (T) BMDMs subjected to *S. aureus* infection for 0, 1, 3, and 6 h yielded transcriptome expression profiles of 80087 BMDMs, of which 34525 cells were from the naive group, and 45562 cells were from the BLP‐trained group (**Figure** **1**A). After quality control, normalization, and data integration processes, we finally obtained a dataset of transcriptome expression profiling of BMDMs with 79596 cells, of which 34270 cells and 45326 cells belonged to the naïve group and the BLP‐trained group, respectively (Table S1, Supporting Information), and 19969 genes. We selected the first 15 principal components for unsupervised clustering analysis and, consequently, 13 distinct subpopulations of BMDMs were identified using the Louvain method.^[^ ^9^ ^]^ As shown in Figure 1B, BMDMs in the same subpopulation clustered together after t‐SNE dimensionality reduction, indicating that t‐SNE and the unsupervised clustering algorithm are consistent in the similarity of cell phenotypes. Figure 1C,D shows the expression of macrophage markers F4/80 and CD11b. The C1‐C10 subpopulations widely expressed *Adgre1* (F4/80) and *Itgam* (CD11b), while the C11, C12, and C13 subpopulations contained very low F4/80^+^ and CD11b^+^ cells (Figure S1A, Supporting Information), accounting for only 1.5%, 0.2%, and 0.1% of the total cell counts (Table S1, Supporting Information). Figure 1E presents a heatmap of differentially expressed genes (DEGs) in each subpopulation of the 13 subpopulations.

To further identify these 13 subpopulations, we conducted in‐depth analysis using myeloid markers and M1/M2 characteristic molecules.^[^ ^10^ ^]^ Notably, none of these 13 subpopulations contained myeloid‐derived suppressor cells (MDSCs) [Cd11c^+^, Gr1(Ly6g, Ly6c)^+^] and CD163^+^ cells (Figure S1B, Supporting Information). Based on the classic M1 (*Cd38*, *Fpr2*, *Tlr2,Gpr18*, *Nos2*, *Ptgs2*, *Il1b*, *Tnf*, *Il6*) and M2 (*Myc*, *Egr2*, *Arg1*, *Mrc1*, *Chil3*, *Retnla*, *Fn1*, *Mgl2*, *Il10*) gene expression profiling, C1, C3, and C6 expressed higher levels of M1 markers, while C2, C12, and C13 expressed relatively low levels of both M1 and M2 markers (Figure S1C,D, Supporting Information). C1, C3, and C6 also highly expressed *Tnf* and *Il1b* (Figure S1F, Supporting Information). By combining specific highly expressed genes in each subpopulation with gene ontology (GO) functional annotation analysis (Table S2, Supporting Information), C1, C3, and C6 were defined as M1. C2, representing the largest subpopulation in the resting status, was defined as M0. C4 and C8 were defined as “intermedium” since ≈50% of cells in C4 and C8 highly expressed M1 markers (Figure S1C, Supporting Information). C5 and C7 emerged as the two main subpopulations after BLP training and were defined as Mta (BLP‐trained macrophage) and Mtb, respectively. C9 was typical dendritic cells (DCs) (CD11b^+^, CD11c^+^) (Figure S1B, Supporting Information),^[^ ^11^ ^]^ and therefore defined as DC‐like macrophages. C10, characterized by high expression of Cd74 and MHC II‐related genes (*H2‐Aa*, *H2‐Ab1*, *H2‐Eb1*, *H2‐DMb1*, *H2‐DMa*), was identified as macrophages with high expression of MHC II (antigen‐presenting macrophages). C11, featuring highly expressed mitochondrial genes (*mt‐Nd3*, *mt‐Co3*, *mt‐Co2*, *mt‐Nd2*) (Figure S1E, Supporting Information), was designated as mitochondria‐rich macrophages.^[^ ^12^ ^]^ C12, characterized by upregulated cell cycle‐related genes (*Birc5*, *Ube2c*, *Ccna2*, *Cenpf*) and enriched cell mitosis‐related GO items, was defined as stem cell‐like macrophages,^[^ ^13^ ^]^ which is identical to the findings by Lin et al.^[^ ^14^ ^]^ C13, characterized by highly expressed genes associated with epithelial cells and cell matrix adhesion (*Sparc*, *Igfbp5*, *Ngfr*, *Col1a2*, *Col1a1*, *Postn*), was defined as epithelial‐like cells (Figure 1E).^[^ ^15^ ^]^ These results suggest that BMDMs in the resting status are composed of multiple subtypes with distinct functional characteristics, and significantly, BLP training induces two specific BMDM subpopulations, namely C5 and C7.

### 2.2 BLP Training Causes Macrophage Inter‐Subpopulation Transformation and Immunity Alterations

**Figure 2:**
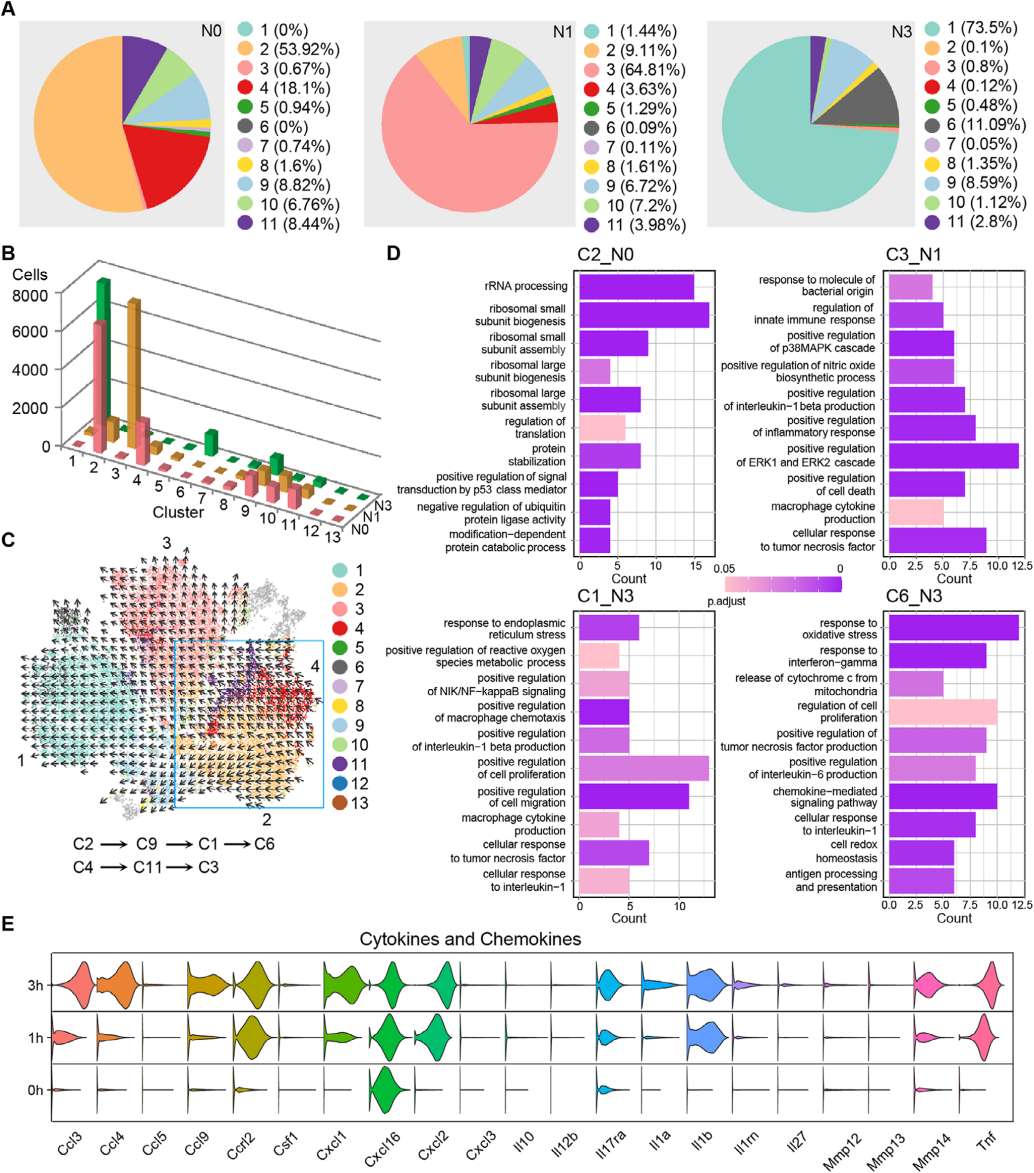
Subpopulation characteristics of naïve macrophages in the resting status and after bacterial infection. A) Cluster distribution was shown as a percentage of total cells at different time points in response to bacterial infection in naïve BMDMs. B) Distribution of naive BMDMs across different clusters and time courses. C) tSNE of cellular trajectories with naive sub‐groups. Cells were colored based on the original clusters in Figure 1B. D) GO BP enrichment analysis of highly upregulated genes (lnFC >0.25) in the comparisons of different time courses in naive conditions. E) Distribution of log‐transformed gene expression of cytokines and chemokines across different time courses in naive conditions. N0, Naïve macrophages without bacterial infection. N1, Naïve BMDMs treated with bacterial for 1 h. N3, Naïve macrophages treated with bacterial for 3 h.

In the resting status, the subpopulations with more than 1% of BMDMs included C2, C4, C8, C9, C10, and C11. Among them, C2 had the highest proportion at 53.9%, followed by C4, C8, C9, and C10 (**Figure** **2**A,B). Upon *S. aureus* infection, there were rapid changes in the cell number and percentage of these subpopulations (Figure 2A,B). At 1 h after infection, C3 became predominant, reaching 64.8%, while at 3 h after infection, C1 and C6 dominated the majority, accounting for 73.5% and 11.1%, respectively. Upon bacterial infection, the overall trend was a reduction in the number of subpopulations, with only 6 subpopulations remaining with cell numbers exceeding 100 at 3 h after infection, and a shift toward M1‐like subtypes C3, C1, and C6 (Figure S2A, Supporting Information). Pseudo‐chronological analysis suggested that BMDM subpopulations differentiated in two directions, namely C2 → C9 → C1 → C6 and C4 → C11 → C3 (Figure 2C). GO enrichment analysis of differential genes revealed distinct functions of major BMDM subpopulations: macrophage cytokine production and IL‐1 regulation for C1, ribosomal biogenesis and ribosome assembly for C2, IL‐1β production and MAPK pathway activation for C3, and monocyte proliferation, oxidative stress response, and cell redox homeostasis for C6 (Figure 2D). Analysis of proinflammatory cytokines and chemokines at different time points showed significant upregulation of *Tnf*, *Mmp14*, *Il1b*, *Il1a*, *Il17ra*, *Cxcl1*, *Cxcl2*, *Ccrl2*, *Ccl3*, *Ccl4*, and *Ccl9* after bacterial infection. Notably, several cytokines at 3 h after infection significantly exceeded the levels at 1 h after infection, indicating an augmented inflammatory response (Figure 2E). At 1 h after infection, the number of DEGs in the naïve group was 249, and GO analysis found that the main functions of these DEGs were related to the response to peptidoglycan, the TLR2 signaling pathway, and regulation of IL‐1 production (Figure S2B and Table S3, Supporting Information). At 3 h after infection, the number of DEGs increased to 557, and in addition to the enhanced above functions, these DEGs were associated with augmented FcγR signaling pathways, chemokine production, cell migration, and positive regulation of podosome assembly (Figure S2B and Table S3, Supporting Information). These results suggest that with prolonged bacterial infection, the inflammatory response of naïve BMDMs continues to increase with enhanced ability in phagocytosis and bacterial elimination.

**Figure 3:**
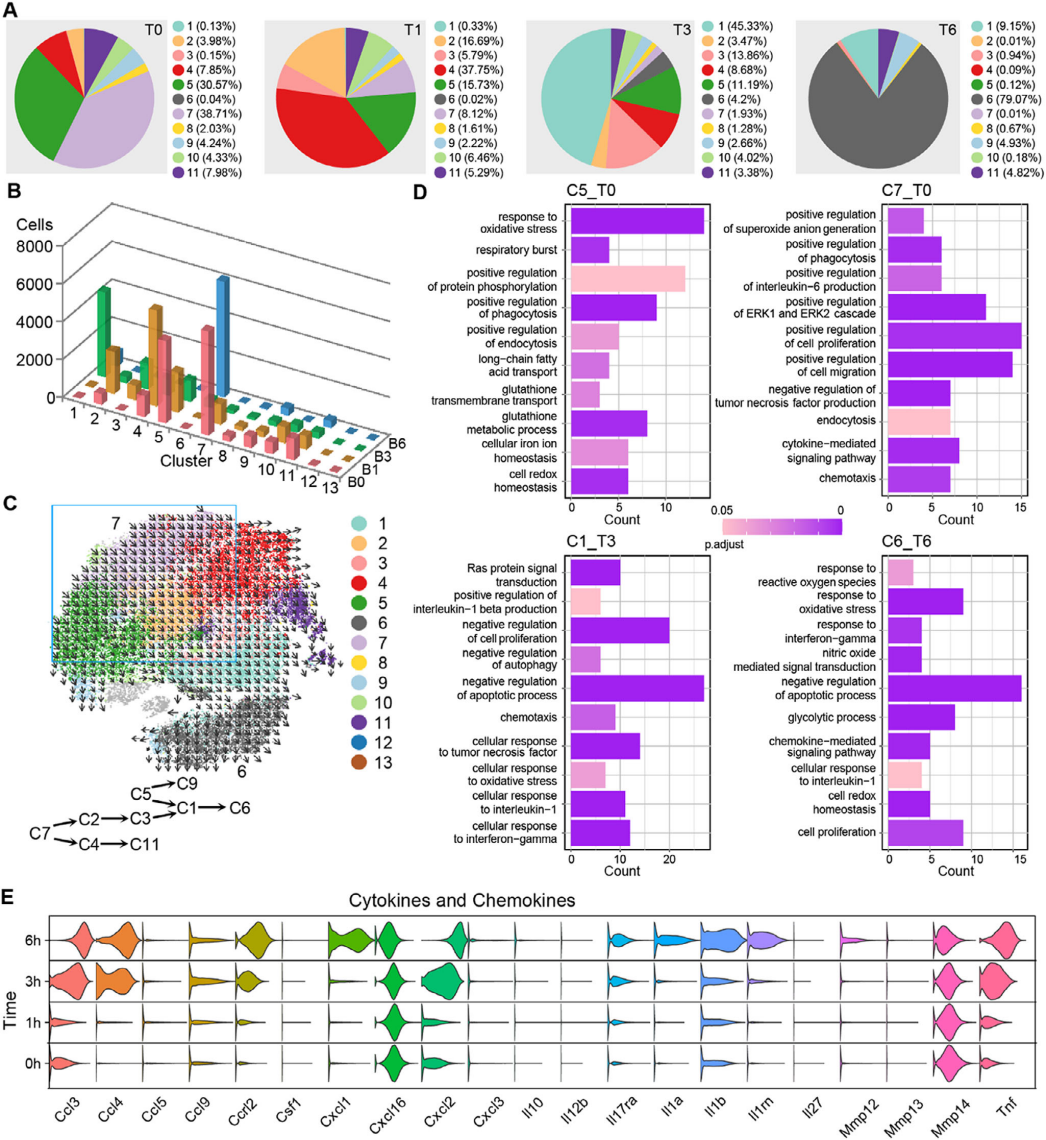
Subpopulation characteristics of BLP‐trained macrophages in the resting status and after bacterial infection. A) Cluster distribution was shown as a percentage of total cells at different time points in response to bacterial infection in the BLP‐trained condition. B) Distribution of BMDMs across different clusters and time courses in BLP‐trained conditions. C) tSNE of cellular trajectories with treated sub‐groups. Cells were colored based on the original clusters in Figure 1B. D) GO BP enrichment analysis of highly upregulated genes (lnFC >0.25) in the comparisons between bacteria‐infected BMDMs and uninfected BMDMs in the BLP‐pretreated condition. Enriched gene numbers are indicated in brackets. E) Distribution of log‐transformed gene expression of cytokines and chemokines across different time courses in the BLP‐trained condition. T0, BLP‐trained BMDMs without bacterial infection. T1–T6, BLP‐trained macrophages with bacteria for 1–6 h.

BMDMs after BLP training consisted of 13 subpopulations, of which C5 (30.57%) and C7 (38.71%) constituted the largest proportions and accounted for 69.28% of total BMDMs before infection, followed by C11, C4, C10, and C9 (**Figure** **3**A,B). Upon *S. aureus* infection, the cell number and percentage of each subpopulation changed rapidly (Figure 3A,B). At 1 h after infection, C4 (37.75%), C2 (16.69%), and C5 (15.73%) were predominant, reaching a total of 70.17%. At 3 h after infection, C1 was the majority, accounting for 45.33%. At 6 h after infection, C6 emerged as the main subpopulation, reaching 79.07% and moreover, the overall number of subpopulations decreased, and only 4 subpopulations were left with more than 100 cells. At the early stage of infection, the distribution of BMDM subpopulations was temporarily dispersed, but as the bacterial infection prolonged, the number of subpopulations decreased and shifted toward the M1‐like subtypes C1 and C6 (Figure S3A, Supporting Information). The Pseudo‐chronological analysis showed that differentiation directions of the BMDM subpopulations in the BLP‐trained group were more complex than those in the naïve group, with four directions, namely C5 → C9, C5 → C1 → C6, C7 → C2 → C3 → C1 → C6, and C7 → C4 → C11 (Figure 3C). C5 and C7, the two unique subpopulations after BLP training, displayed primary functions identified by GO analysis as the response to oxidative stress, glutathione metabolic process, and positive regulation of phagocytosis for C5, and positive regulation of cell proliferation, migration, ERK1, and ERK2 cascade for C7. Upon bacterial infection, C1 and C6 became the main subpopulations in BLP‐trained BMDMs. GO analysis showed that the main functions of DEGs in C1 included negative regulation of apoptosis and cell proliferation, while the main functions of DEGs in C6 were associated with responses to both oxidative stress and interferon‐gamma (IFN‐γ) in addition to negative regulation of apoptosis (Figure 3D). Analysis of macrophage‐related cytokines and chemokines found that *Tnf*, *Il1b*, *Il1a*, *Ilrn*, *Il17ra*, *Cxcl1*, *Cxcl2*, *Ccrl2*, *Ccl3*, *Ccl4*, and *Ccl9* expression significantly increased over the course of infection (Figure 3E). At the early stage of infection, BLP‐trained BMDMs displayed fewer DEGs (Figure S3B and Table S4, Supporting Information); however, as the infection progressed, the number of DEGs continued to increase and reached 428 at 6 h after infection (Table S4, Supporting Information). BLP‐trained BMDMs at 3 h after infection exhibited a much stronger response to bacteria‐derived pathogen‐associated molecular patterns (PAMPs) than that at 1 h after infection, while more active regulations of cytokine production, reactive oxygen metabolism, and leukocyte migration were observed in BLP‐trained BMDMs at 6 h after infection compared with those at 3 h after infection (Figure S3B, Supporting Information).

### 2.3 Characterization of Functionally Enhanced BLP‐Trained Macrophage Subpopulations

**Figure 4:**
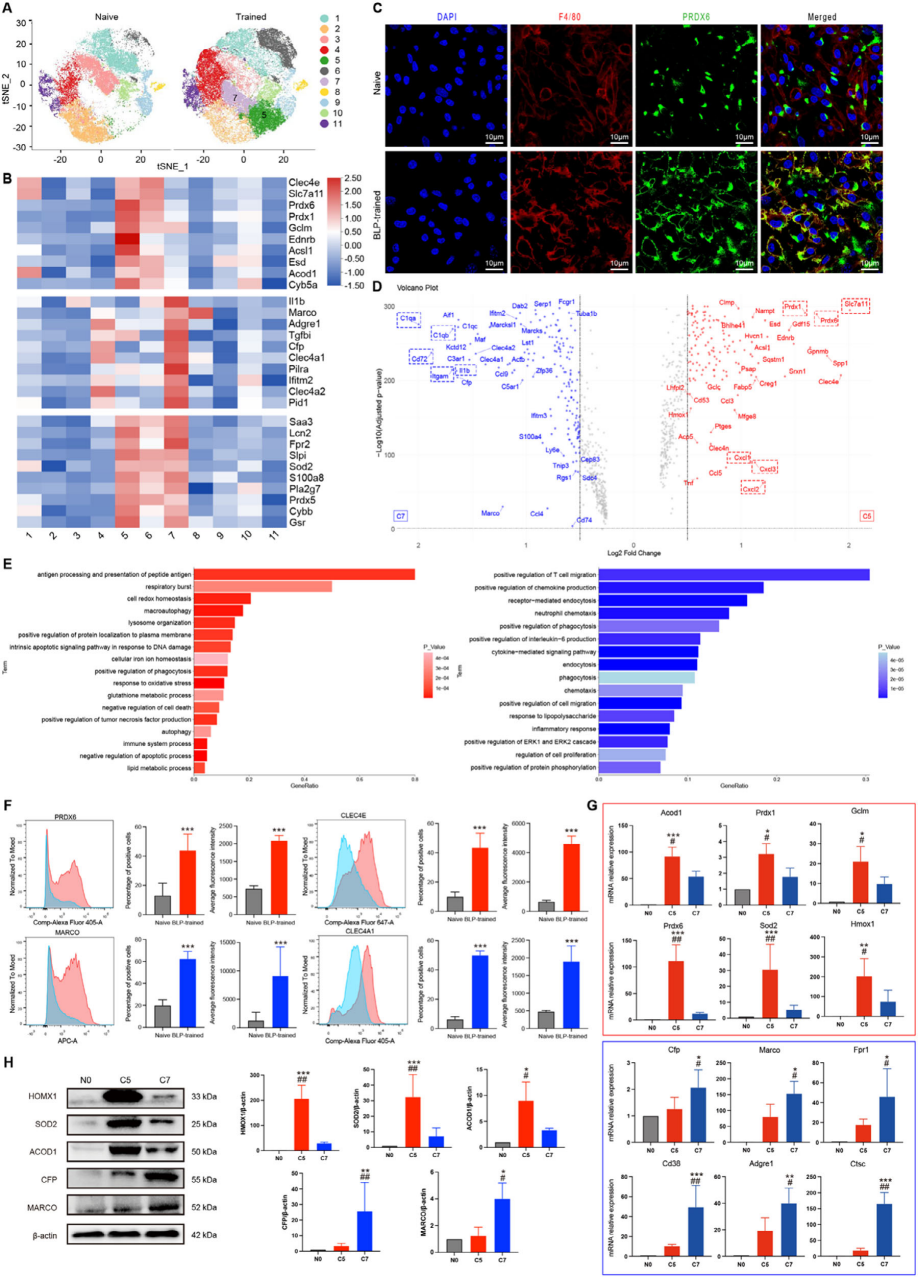
Two specific subpopulations induced by BLP‐trained tolerance and their characteristics. A) Split tSNE plot by naïve and BLP‐trained BMDMs based on gene expression. B) Heatmaps of markers of C5, C7, and both C5 and C7. C Immunofluorescence analysis of PRDX6 (green) and F4/80(red) (n = 3 independent experiments). D) Volcano Plot showing differential genes for C5 and C7. (*p*< 0.05, logFC >0.585). E) KEGG enrichment analysis of highly upregulated genes (logFC >0.585) between C5 and C7. F) Flow cytometry peak charts, positive cell ratios, and average fluorescence intensities for PRDX6, CLEC4E, MARCO, and CLEC4A1 in naïve and BLP‐trained BMDMs. G) Expression of Acod1, Prdx1, Gclm, Prdx6, Sod2, Hmox1, Cfp, Marco, Fpr1, Cd38, Adgre1 and Ctsc mRNA in naïve, C5, and C7 BMDMs was assessed by RT‐qPCR. Results are the mean ± SEM of three independent experiments. H) Expression of HOMX1, SOD2, ACOD1, CFP, and MARCO proteins in naive, C5, and C7 BMDMs was assessed by Western blot analysis. ^*^ *p*< 0.05 compared with naive BMDMs, ^#^ *p*< 0.05 vs the alternative BMDM subtype (i.e., C5 compared with C7, or C7 compared with C5); n = 3 independent experiments.

By comparing the cell number in each subpopulation, we observed that the total cell counts in C4, C5, C6, and C7 in the BLP‐trained group were greater than those in the naïve group, while the total cell counts in C1, C2, C3, and C9 in the naïve group were higher than those in the BLP‐trained group. Notably, C5 and C7 were the dominant subpopulations in the BLP‐trained group (Table S1, Supporting Information) and, more importantly, both emerged as specifically differentiated subpopulations by BLP training (**Figure** **4**A). To understand the phenotypic characteristics of C5 and C7, we first identified distinctively expressed molecules from DEGs in these two subpopulations (Table S5, Supporting Information). Figure 4B shows the top‐10 specific genes in C5 and C7. Most of the C5‐specific genes were mainly related to antioxidative stress (*Slc7a11*, *Prdx6*, *Prdx1*, *Gclm*) and anti‐inflammatory (*Clec4e*, *Acod1*), suggesting that C5 is the main subpopulation in mediating anti‐inflammatory and antioxidative stress effects by BLP training,^[^ ^16^ ^]^ whereas most of the C7‐specific genes were predominantly associated with phagocytosis, bactericidal effect (*Marco*, *Cfp*), and the regulation of immune homeostasis (*Clec4a1*, *Clec4a2*, *Ifitm2*).^[^ ^17^, ^18^ ^]^ Interestingly, we found that the shared DEGs in C5 and C7 were mostly associated with anti‐inflammatory (*Lcn2*, *Fpr2*, *Slpi*) and antioxidative stress (*Sod2*, *Prdx5*, *Gsr*), suggesting that anti‐inflammatory and antioxidative stress are the main functional characteristics of BLP‐trained BMDMs (Figure 4B). We then examined the protein expression and cellular distribution of PRDX6, an important molecule closely associated with antioxidative stress response, and compared them between BLP‐trained BMDMs and naïve BMDMs. As shown in Figure 4C and Figure S4A,B (Supporting Information), immunofluorescent analysis showed substantially increased expression of PRDX6 and its co‐localization with the membrane F4/80 in BLP‐trained BMDMs at 24 h after BLP training, and moreover, the increased PRDX6 expression observed in BLP‐trained BMDMs was mainly distributed on the cell membrane, which was in sharp contrast to its distribution around the nucleus seen in naïve BMDMs.

To further clarify the functional characteristics of C5 and C7, we analyzed highly expressed genes in C5 vs C7 (Table S6, Supporting Information). Notably, C5 highly expressed antioxidant genes (*Slc7a11*, *Prdx1*, *Prdx6*) and chemokine genes (*Cxcl1*, *Cxcl2*, and *Cxcl3*) when compared to C7,^[^ ^16^, ^19^ ^]^ while C7 mainly expressed M1 polarization markers (*C1qa*, *C1qb*, *Cd72*) and proinflammatory genes (*Il1b*, *Itgam*) when compared to C5 (Figure 4D).^[^ ^7^, ^20^ ^]^ Kyoto Encyclopedia of Genes and Genomes (KEGG) pathway enrichment analysis found that the highly expressed genes in C5 were mainly related to biological processes such as antigen presentation, respiratory burst, cell redox homeostasis, and macroautophagy, whereas the highly expressed genes in C7 were mostly linked to positive regulation of T cell migration, neutrophil chemotaxis, and phagocytosis (Figure 4E; Table S6, Supporting Information).

To validate the above analysis of the phenotypic characteristics of C5 and C7 subpopulations, the differentially expressed molecules on the membrane surface of BMDMs, with PRDX6 and CLEC4E as the surface markers for C5, and MARCO and CLEC4A1 as the surface markers for C7, were analyzed by FACScan analysis (Figure 4F). The results showed that the number of cells with high expression of PRDX6, CLEC4E, MARCO, and CLEC4A1, as well as the intensity of expression, were significantly increased and enhanced in BLP‐trained macrophages compared with naive cells. Based on this, we defined the PRDX6‐high‐expression cell population (≈30%) and the MARCO‐high‐expression cell population (≈30%) as C5 and C7 macrophages, respectively (Figure 4F, Figure S4C,D, Supporting Information), and isolated these two cell populations by FACSan sorting for further detection. Real time quantitative RT‐PCR (RT‐qPCR) results showed that compared with the C7 subcluster, C5 macrophages highly expressed anti‐inflammatory and antioxidant genes Acod1, Prdx1, Gclm, Prdx6, Sod2, and Hmox1, whereas C7 macrophages had upregulated expression of genes involved in phagocytosis and bactericidal activity, including Cfp, Marco, Fpr1, Cd38, Adgre1, and Ctsc in comparison with the C5 subcluster (Figure 4G). Notably, these genes were all expressed at much lower levels in untrained naive macrophages, suggesting that the C5 subpopulation, which highly expresses anti‐inflammatory and antioxidant stress genes, and the C7 subpopulation, which highly expresses phagocytic and bactericidal genes, are unique cell subpopulations induced by BLP training. Western blotting results showed elevated expression of HO‐1, SOD2, and ACOD1 in the C5 subpopulation, while CFP and MARCO were enriched in the C7 subpopulation (Figure 4H), indicating that protein levels of these molecules were consistent with their transcriptional levels. These results reveal the heterogeneity of macrophages for the first time and identify two subpopulations within BLP‐trained macrophages with distinct functional characteristics directly related to their protective functions.

### 2.4 The Transcriptional Mechanism of Altered BLP‐Trained Macrophage Immunity

**Figure 5:**
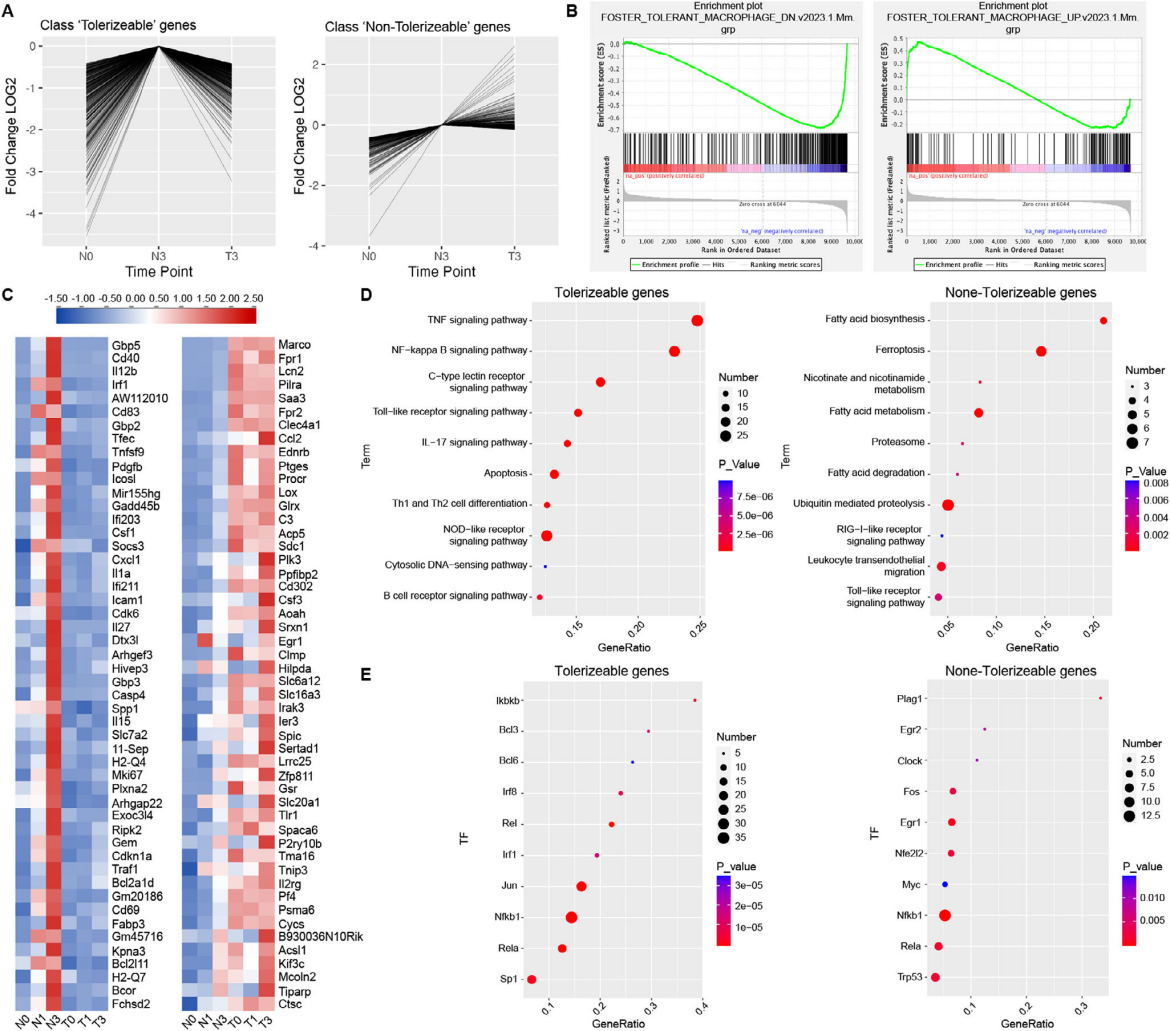
Identification of expression patterns of T‐gene and NT‐gene in BLP‐trained macrophages. A) Expression fold plots of the T and NT genes; all values in the plots are in log10 of the control value for N3 expression of the genes. B) GESA enrichment analysis of the T gene and NT gene. C) Heatmap showing the top 50 T genes and NT genes. D) TOP10 pathways of the T genes and the NT genes were enriched using KEGG. E) TOP10 transcription factors regulating T genes or NT genes were predicted using TRRUST. T‐gene: tolerizeable gene; NT‐gene: non‐tolerizeable gene.

To reveal the transcriptional mechanisms behind BLP‐trained macrophage immunity, two distinct gene expression patterns in macrophages, namely tolerizeable genes (T genes), which are not inducible in tolerant macrophages, and non‐tolerizeable genes (NT genes), which are inducible in tolerant macrophages, were analyzed.^[^ ^21^ ^]^ In the present study, we found that BLP‐trained BMDMs displayed similar gene expression patterns (**Figure** **5**A), and moreover, gene set enrichment analysis (GSEA) revealed a high matching degree in T genes between BLP‐trained BMDMs and LPS‐trained macrophages (Figure 5B); however, some LPS NT genes (*Cd200*, *Slc25A37*, *Elk3*) functioned as T genes in BLP‐trained BMDMs (Table S7, Supporting Information). Figure 5C and Table S7 (Supporting Information) illustrate the top‐50 T genes (461) and NT genes (221) in BLP‐trained BMDMs. Among the top‐50 T genes, 32 genes were associated with the proinflammatory response (*Cd40*, *Il12b*, *Irf1*, *etc*.),^[^ ^22^ ^]^ 18 genes demonstrated involvement in promoting reactive oxygen species (ROS) generation (*Gadd45b*, *Cxcl1*, *Icam1*, etc.),^[^ ^23^, ^24^, ^25^ ^]^ and additionally, a distinct subset appeared related to innate immunocyte tolerance (*Tfec*, *Socs3*, *Il27*, etc.).^[^ ^26^, ^27^, ^28^ ^]^ Conversely, among the top‐50 NT genes, 22 genes were related to the anti‐inflammatory response (*Pilra*, *Saa3*, *Clec4a1*, etc.),^[^ ^29^, ^30^, ^31^ ^]^ 17 genes were related to oxidative stress resistance (*Glrx*, *Csf3*, *Aoah*, etc.),^[^ ^32^, ^33^, ^34^ ^]^ and 25 genes were related to innate immunocyte tolerance (*Marco*, *Fpr1*, *Lcn2*, etc.).^[^ ^21^ ^]^ KEGG pathway enrichment analysis found that T genes in BLP‐trained BMDMs predominantly clustered in the inflammatory response pathway (Figure 5D; Table S7, Supporting Information). Intriguingly, several fatty acid metabolism pathways for fatty acid biosynthesis, metabolism, and degradation, and the ferroptosis pathway emerged prominently in the top‐10 enrichment pathways of NT genes in BLP‐trained BMDMs (Figure 5D; Table S7, Supporting Information), suggesting a potential link between BLP‐trained tolerance and ferroptosis. Transcriptional regulatory relationships unraveled by sentence‐based text‐mining (TRRUST) analysis of the upstream transcription factors in BLP‐trained BMDMs found that T genes were mostly enriched in transcription factors related to mediating inflammation and apoptosis (*Irf1*, *Nfkb1*, *Bcl3*, *Bcl6*) (Figure 5E; Table S7, Supporting Information),^[^ ^35^, ^36^ ^]^ while NT genes were predominantly enriched in transcription factors associated with regulating phagocytosis (*Plag1*), cell differentiation and development (*Myc*, *Fos*, *Egr1*) (Figure 5E; Table S7, Supporting Information).^[^ ^37^, ^38^, ^39^ ^]^ Notably, *Nfe2l2* (NRF2), a crucial transcription factor in resisting oxidative stress,^[^ ^40^ ^]^ was also highly enriched in NT genes (Figure 5E; Table S7, Supporting Information), suggesting a pivotal role for NRF2 in the regulation of NT gene expression for BLP‐trained tolerance against oxidative stress.

LPS‐trained tolerance is a classical model for studying the negative feedback mechanism in inflammation, and its protective effects in sepsis and infection have been extensively studied.^[^ ^41^ ^]^ Similar to LPS, BLP is also a PAMP molecule; however, it activates distinct signaling pathways. To explore the differences between LPS‐trained tolerance and BLP‐trained tolerance, we analyzed the transcriptome data of LPS training from the publicly available dataset GSE8621 and compared it with our transcriptome data of BLP training. Although the secondary stimulus in GSE8621 was LPS, whereas in our study it was *S. aureus*, the inflammatory response induced by LPS or *S. aureus* was comparable, thus allowing a meaningful comparative analysis (Figure S5A, Supporting Information).

Given that the main feature in both LPS‐trained tolerance and BLP‐trained tolerance is the enhanced anti‐inflammatory responses, we first examined the expression levels of inflammatory mediators after secondary stimulation in LPS‐trained or BLP‐trained macrophages. These inflammatory tolerance genes were categorized into five distinct groups (Figure S5B, Supporting Information). Although both BLP and LPS training significantly suppressed the transcription of key inflammatory genes such as *Nfkb1*, *Il18*, *IL17ra*, *Ccl6*, and *Cxcl10* (in orange) upon secondary stimulation, BLP training demonstrated a much stronger ability to downregulate certain genes such as *Nfkbiz* (a known regulator of itaconate‐mediated anti‐inflammatory pathways), *Tnf*, *Nfkb2*, and *Il12b* (in yellow), whereas LPS training more effectively suppressed *Il10*, *Il6*, *Ccr5*, and *Ccrl2* (in red). Notably, certain inflammatory genes, including *Tlr2*, *Il12a*, and *Il16* (in green) as well as *Tlr1*, *Tlr6*, and *Il3ra* (in blue), were markedly downregulated in BLP‐trained but not in LPS‐trained macrophages, where they either remained unchanged or were even upregulated. Conversely, genes such as *Tlr4*, *Ccl2*, *Ccl12*, *Ccr3*, and *Cxcl4* (in purple) were significantly downregulated in LPS‐trained but not in BLP‐trained macrophages. Consistent with previous studies, despite common features among different TLR ligand‐induced tolerance states, their protective roles appear to differ.^[^ ^42^ ^]^ Of particular interest, we found evidence suggesting a degree of “specificity” in TLR ligand‐induced innate immune memory, for instance, TLR4 ligand, i.e., LPS‐trained tolerance, leads to downregulation of *Tlr4* and upregulation of *Tlr1/2* and *Tlr2/6*, whereas TLR2 ligand, i.e., BLP‐trained tolerance, results in upregulation of *Tlr4*. Moreover, BLP‐trained macrophages exhibited enhanced chemokine production (in purple). Finally, we compared the gene expression profiles between BLP‐trained and LPS‐trained macrophages. Among the DEGs, the largest overlap was observed in the NT gene set, comprising 3,503 shared genes between BLP training and LPS training (Figure S5C, Supporting Information), whereas a total of 103 T genes were common to both training conditions. In terms of training condition‐specific genes, 173 NT genes were uniquely associated with BLP training, while 124 T genes were specific to LPS training. Furthermore, 173 T genes were reversed by BLP training, and 22 NT genes were reversed by LPS training. A detailed list of these gene sets is provided in Table S8 (Supporting Information).

### 2.5 A Novel Mechanism of BLP Training–A Role for NRF2 in Reducing Ferroptosis

**Figure 6:**
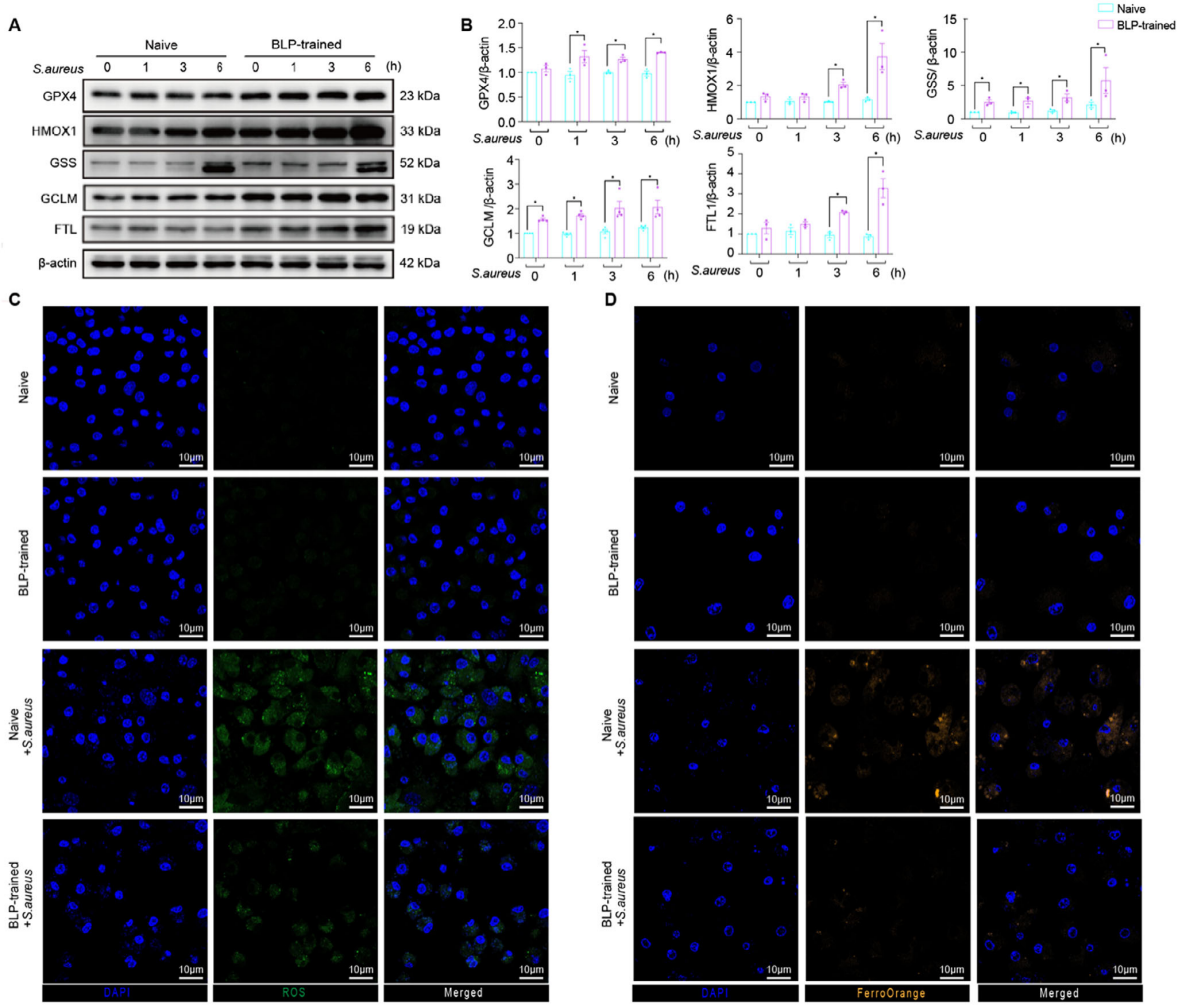
BLP‐trained tolerance attenuates *S. aureus* infection‐initiated ferroptosis. A) Expression of GPX4, FTL, GSS, HMOX, and GCLM protein in naive and BLP‐trained BMDMs at the indicated time points after *S. aureus* stimulation was assessed by Western blot (n = 3–4 independent experiments). B) Bands corresponding to signals of proteins (A) were scanned and analyzed. The intensity of each band was corrected by its corresponding Actin band and expressed as the ratio of the intensity detected in the unstimulated macrophages. The results shown represent one experiment from a total of three separate experiments. All data are presented as the mean ± SEM of three separate experiments. ^*^ *p*< 0.05 compared to naive BMDMs at the same time point as bacterial infection. C,D) Confocal imaging of ROS (green), FerroOrange (orange), and DAPI (blue) in naive macrophages and BLP‐trained macrophages with *S. aureus* infection. The results shown represent one experiment from a total of three separate experiments. Cell nuclei were stained with DAPI. Scale bar = 10 µm.

Previous studies mostly focused on T gene‐associated tolerant effects; however, accumulated evidence in recent years has highlighted the significance of NT genes in tolerance regulation.^[^ ^43^, ^44^, ^45^ ^]^ In the present study, we analyzed DEGs in BLP‐trained tolerance and found that NT genes were significantly clustered in the ferroptosis pathway (Figure 5D). Utilizing the single‐cell transcriptomic data from naïve and BLP‐trained BMDMs combined with KEGG mapper, we constructed a proportional map of ferroptosis pathway‐related gene expression, and as shown by the map, glutathione synthesis‐related enzymes *Gss*, *Gclm*, and *Gclc* ^[^ ^46^ ^]^ were substantially upregulated, while enzymes *Steap3* and *Ncoa4* involved in the conversion of Fe^3+^ into Fe^2+[^ ^47^ ^]^ were downregulated in BLP‐trained BMDMs (Figure S6A, Supporting Information). Western blot analysis showed that compared with naive BMDMs, BLP‐trained BMDMs exhibited significantly increased GSS, GCLM, and GPX4 both before infection and 1–6 h after infection (**Figure** **6**A,B). FTL, which participates in iron ion transport and storage, and HMOX1, which maintains cellular redox homeostasis, were also increased in BLP‐trained BMDMs (Figure 6A,B). Immunofluorescent staining demonstrated that upon *S. aureus* infection, naïve BMDMs exhibited increased ROS levels and accumulated iron ions, whereas BLP‐trained BMDMs displayed relatively low ROS levels (Figure 6C; Figure S6B, Supporting Information) and reduced iron ions (Figure 6D; Figure S6C, Supporting Information). These results suggest that BLP‐trained BMDMs can resist bacterial infection‐initiated ferroptosis by enhancing glutathione synthesis, maintaining redox homeostasis, attenuating ROS generation, and simultaneously facilitating iron ion transport and storage to prevent iron accumulation.

**Figure 7:**
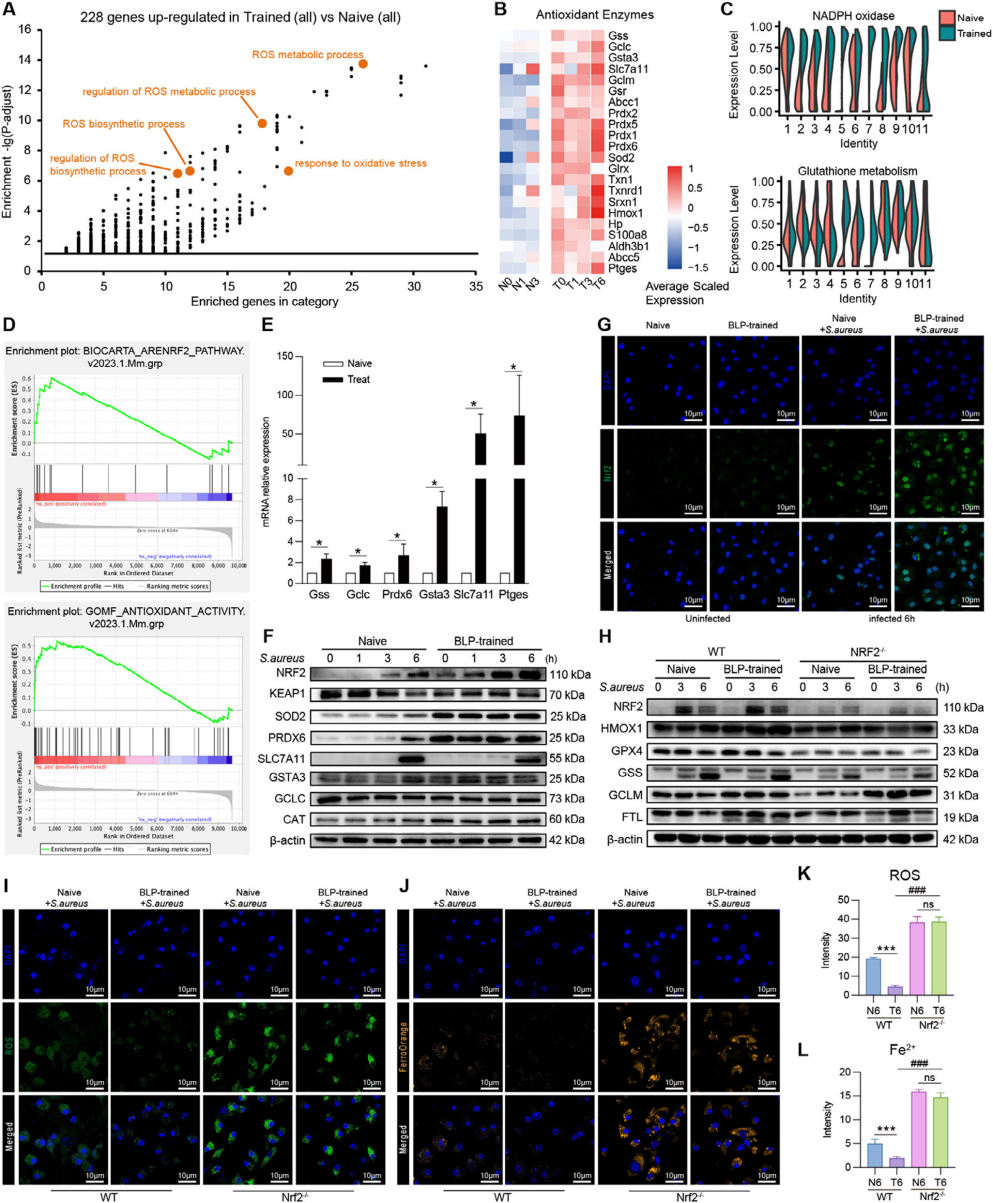
BLP‐trained tolerance attenuates *S. aureus* infection‐initiated ferroptosis by augmenting the antioxidative stress capability via activation of NRF2. A) Gene Ontology enrichment analysis of biological processes in 228 genes up‐regulated in trained tolerance vs naive BMDMs. Emphasized circles are biological processes related to ROS biosynthetic and metabolic processes. B) Heatmap of genes encoding antioxidant enzymes across different treatment and time courses. C) Violin plots demonstrating the expression of NADPH oxidase and glutathione metabolism‐related genes in different groups in Naive and BLP‐trained groups. D) Anti‐oxidative pathways (Nrf2, antioxidant activity) were analyzed using GSEA in the T3 compared to the N3 group. E) Expression of *Gss*, *Gclc*, *Prdx6*, *Slc7a11*, and *Ptge* mRNA in naïve and BLP‐trained BMDMs was assessed by RT‐qPCR. Results are the mean ± SEM of three independent experiments. ^*^ *p*< 0.05 compared with naive BMDMs (n = 4–6 independent experiments). F) Expression of NRF2, KEAP1, SOD2, PRDX6, SLC7A11, GSTA3, GCLC, and CAT protein in naive and BLP‐trained BMDMs at the indicated time points after *S. aureus* stimulation was assessed by Western blot analysis (n ≥ 3 independent experiments). G) Confocal images were taken after naive and BLP‐trained BMDMs were subjected to *S. aureus* for 6 h infection by immunofluorescent staining with the anti‐Nrf2 Ab (green) and Alexa Flour 594‐conjugated secondary Ab. Cell nuclei were stained with DAPI (blue) (n = 3 independent experiments). H) Cytoplasmic proteins of NRF2, HMOX1, GPX4, GSS, GCLM, and FTL in naïve and BLP‐trained BMDMs from *Nrf2* ^−/−^ or wide type mice were assessed by Western blot analysis (n ≥ 3 independent experiments). I,J) Confocal imaging of ROS (green) and FerroOrange (orange) was taken after naïve and BLP‐trained BMDMs from *Nrf2* ^−/−^ or wide type mice were subjected to *S. aureus* infection for 6 h. Cell nuclei were stained with DAPI (blue). Scale bar = 10 µm. K, L Fluorescence Intensity Analysis of I, J.

To further investigate the molecular mechanism(s) by which BLP‐trained BMDMs resist ferroptosis, we analyzed the influence of BLP training on cellular oxidative stress‐related pathways. Compared with the naive BMDMs, BLP‐trained BMDMs displayed augmented ROS generation, metabolism, and regulation as indicated by single‐cell transcriptomic data (**Figure** **7**A) as well as upregulated antioxidative stress kinases before and after infection, as shown by gene heatmap (Figure 7B). Violin plot analysis revealed that the oxidative stress‐related nicotinamide adenine dinucleotide phosphate (NADPH) oxidase pathway was significantly upregulated in all subpopulations of the BLP‐trained group, and particularly glutathione metabolism‐related pathways were elevated in C5 and C7 of the BLP‐trained group (Figure 7C). Given that *Nfe2l2* (NRF2), the most important transcription factor involved in antioxidative stress, was identified among the enriched NT genes (Figure 5E), we introduced the DEGs obtained from the naïve and BLP‐trained BMDMs into the NRF2 pathway and antioxidative stress pathway using GSEA and found that at 3 h after infection, these DEGs were positively correlated with both pathways (Figure 7D). We next verified the expression of NRF2‐regulated genes at both the mRNA and protein levels, and found that compared with naive BMDMs, *Gss*, *Gclc*, *Prdx6*, *Csta3*, *Slc7a11*, *and Ptge* mRNA levels and NRF2, SOD2, PRDX6, SLC7A11, GSTA3, and CAT protein levels were significantly increased in BLP‐trained BMDMs, while the NRF2 inhibitory protein Keap1 was substantially decreased in BLP‐trained BMDMs (Figure 7E,F; Figure S7A,B, Supporting Information). Immunofluorescent staining further confirmed that the number of cells exhibiting NRF2 nuclear translocation after *S. aureus* infection in the BLP‐trained BMDMs was much higher than that in naive BMDMs (Figure 7G). To explore the regulatory role of NRF2 in BLP training‐associated antioxidative stress response and its correlation with the resistance to ferroptosis, we used *Nrf2* ^−/−^ BMDMs isolated from *Nrf2* ^−/−^ mice. Compared with *Nrf2* ^+/+^ BMDMs, BLP‐trained *Nrf2* ^−/−^ BMDMs displayed downregulated expression of GPX4, HMOX1, GSS, and FTL as revealed by Western blot analysis (Figure 7H), enhanced both intracellular ROS (Figure 7I,K), and ferrous ion (Figure 7J,L) levels as indicated by fluorescent staining, suggesting a crucial role for NRF2 in the process of BLP training to tolerate oxidative stress and to resist bacterial infection‐initiated ferroptosis.

To further determine whether the patterns of antioxidant stress resistance and ferroptosis regulation are unique to BLP training, we compared the transcriptomic profiles between BLP‐trained macrophages and LPS‐trained macrophages. We found that 24 h post stimulation, BLP‐trained macrophages exhibited significantly higher expression levels of both antioxidant and anti‐ferroptosis proteins (except for haptoglobin, Hp) compared to LPS‐trained macrophages, although LPS‐trained macrophages also showed significant upregulation of these proteins in relative to naive controls (Figure S5D, Supporting Information). Moreover, following secondary stimulation, BLP‐trained macrophages demonstrated a pronounced upregulation of anti‐ferroptosis proteins and downregulation of pro‐ferroptosis proteins compared to their LPS‐trained counterparts (Figure S5E, Supporting Information).

### 2.6 BLP‐Trained Macrophages Reprogram Metabolism to Preserve a Protective Intracellular Environment

**Figure 8:**
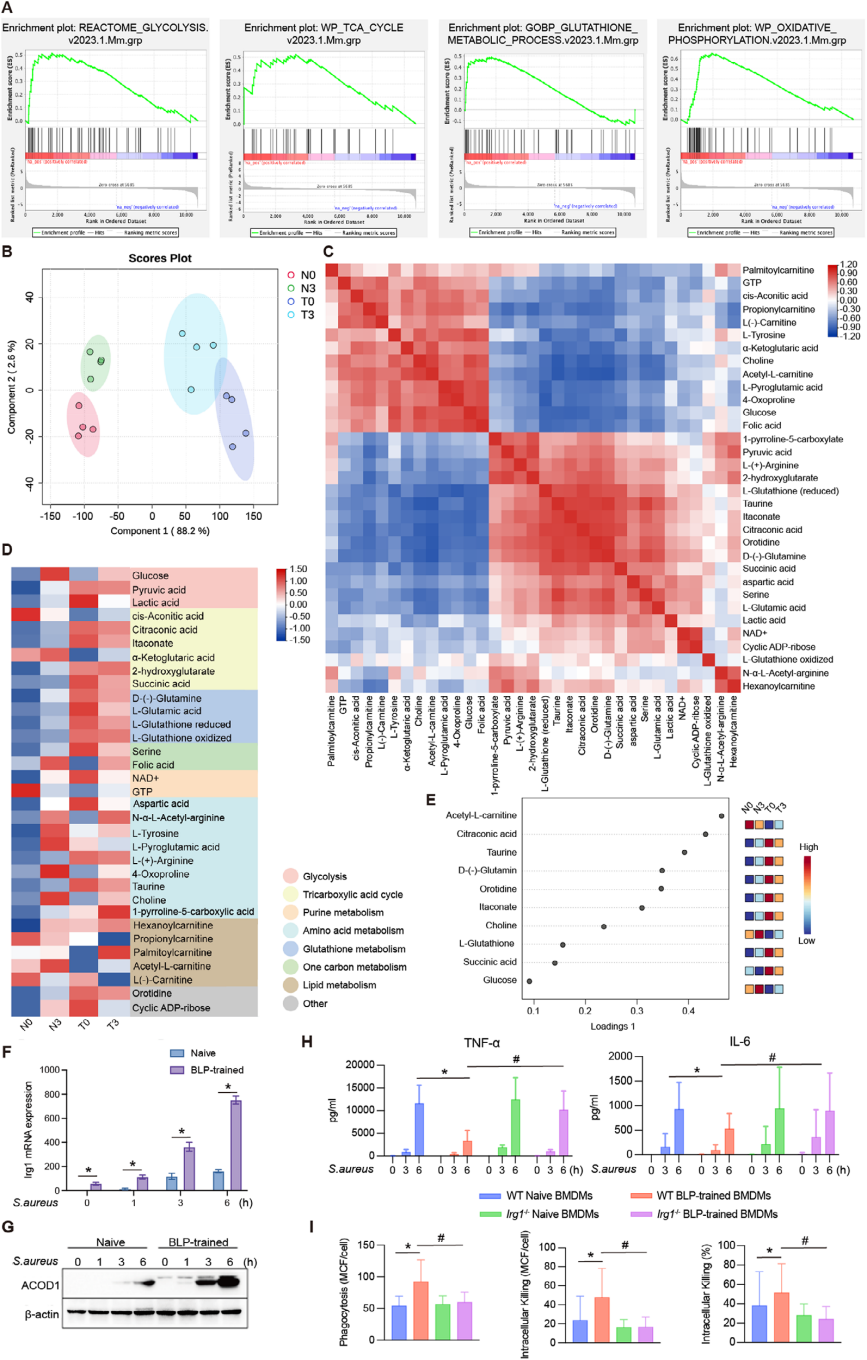
BLP‐trained macrophages undergo metabolic reprogramming. A) Metabolism‐related pathways (glycolysis, tricarboxylic acid cycle, oxidative phosphorylation, glutathione metabolism) were analyzed using GSEA in T3 compared to the N3 group. B) Partial least squares‐discriminant analysis (PLS‐DA) score plot based on all differential metabolites for N0 (red dots), N3 (green dots), T0 (blue dots), and T3 (light blue dots) BMDMs (n = 4 independent experiments). C) Heatmap showing the correlation between metabolites by Person correlation analysis. D) Heatmap showing all statistically significant metabolites in N0, N3, T0, and T3. E) The top 10 metabolites are based on the PLS‐DA scoring model, and the colored box on the right indicates the relative abundance of metabolites in each group of cells. F,G) The mRNA transcription and protein expression levels of Irg1/ACOD1 in the naïve and BLP‐trained BMDMs were measured at 0, 1, 3, and 6 h after *S. aureus* infection (n = 3 independent experiments). H) Proinflammatory cytokines TNF‐α and IL‐6 levels in the supernatant of naïve and BLP‐trained BMDMs isolated from wide‐type (WT) and IRG1‐deficient (*Irg1* ^−/−^) mice at 0, 3, and 6 h after *S. aureus* infection (n = 4 independent experiments). I) Phagocytosis at 30 min and intracellular killing at 60 min of naïve and BLP‐trained WT and *Irg1* ^−/−^ BMDMs after *S. aureus* infection (n = 6 independent experiments). ^*^ *p*< 0.05 vs naive BMDMs, ^#^ *p*< 0.05 vs *Irg1* ^−/−^ BMDMs. N0 and T0, naïve and BLP‐trained BMDM. N3 and T3, naïve or BLP‐trained BMDM, were subjected to *S. aureus* for 3 h.

Numerous studies have demonstrated that metabolic reprogramming is an important foundation for macrophages to augment their anti‐inflammatory capability and resistance to oxidative stress.^[^ ^48^, ^49^, ^50^ ^]^ Therefore, we utilized single‐cell transcriptomic data to analyze the cellular metabolic pathways between naïve and BLP‐trained BMDMs and found that at 3 h after *S. aureus* infection, glycolysis, TCA cycle, oxidative phosphorylation, and glutathione metabolism were significantly enhanced in BLP‐trained BMDMs compared with naïve BMDMs (**Figure** **8**A). Glycolysis‐associated *Ldha*, *Pkm2*, *Hif‐a*, and TCA cycle‐related *Mdh2*, *Acod1* showed higher expression in BLP‐trained BMDMs than in naïve BMDMs (Figure S8A, Supporting Information), which were further validated by RT‐qPCR (Figure S8B, Supporting Information). These results suggest that BLP‐trained BMDMs undergo metabolic reprogramming and have much stronger capabilities in both glycolysis and the TCA cycle.

To corroborate the occurrence of metabolic reprogramming in BLP‐trained BMDMs, we conducted a metabolomic analysis in naïve and BLP‐trained groups. We first analyzed the quality of the metabolomic data obtained using the sparse partial least squares regression. Our results indicated good repeatability within each group, as supported by the proximity of the four replicates. Moreover, there was a clear separation between samples obtained from naïve and BLP‐trained groups before infection and at 3 h after infection, denoting the difference existing between the compared groups and metabolomic profiles (Figure 8B). Correlation and heatmap analyses of metabolomic data revealed that anti‐inflammatory and antioxidative stress metabolites, including itaconate, oxidized and reduced forms of glutathione, and 2‐hydroxyglutarate, were clustered together (Figure 8C). Heatmap analysis further showed that metabolites of glycolysis and the TCA cycle (except α‐ketoglutarate) were significantly elevated in the BLP‐trained group, indicating that BLP‐trained BMDMs display much stronger glycolysis and TCA cycle than naïve BMDMs, which is consistent with the results of single‐cell transcriptomic data analysis (Figure 8D). We then assessed metabolites with the greatest differences across groups using Loading1 scores. Interestingly, itaconate and citraconic acid, a homolog of itaconate, had higher scores, suggesting a pivotal role they play in BLP training (Figure 8E). Finally, we integrated single‐cell RNA‐seq with metabolomic data obtained from the naïve and BLP‐trained groups and observed congruent results, that is, BLP‐trained BMDMs showed an increased ability both in glycolysis, as evidenced by upregulated *Slc2a1*, *Hk2*, *Ldha* expression, and increased pyruvate, lactate levels, and in the TCA cycle, as indicated by upregulated *Aco2*, *Ogdh*, *Suclg1*, *Sdhb*, *Fh1*, *Mdh2* expression and increased citric acid, succinic acid, malic acid, and oxaloacetate levels (Figure S8C, Supporting Information). Notably, substances associated with anti‐inflammatory properties, including itaconate, citraconic acid, 2‐hydroxyglutarate, and metabolites with antioxidative stress ability, including itaconic acid, glutathione, were also substantially increased (Figure S8C, Supporting Information).

To dissect the causal relationship between BLP training‐mediated metabolic reprogramming and macrophage functional phenotypes, we focused on the immunometabolic enzyme IRG1/ACOD1 (responsible for itaconate production) to investigate its role in the anti‐inflammatory and antibacterial effects of BLP‐trained macrophages. First, by using RT‐qPCR and Western blot analysis, we observed that BLP‐trained macrophages exhibited significantly higher mRNA (Figure 8F) and protein (Figure 8G) expressing levels of ACOD1 at 0, 1, 3, and 6 h post *S. aureus* infection in comparison with naïve macrophages, which is consistent with our single‐cell RNA‐seq findings. Second, by using IRG1‐deficient (*Irg1* ^−/−^) macrophages, we demonstrated that the BLP training‐initiated anti‐inflammatory effect, as represented by substantially attenuated TNF‐α and IL‐6 release upon *S. aureus* infection, was almost completely abolished in *Irg1* ^−/−^ macrophages (Figure 8H). Finally, while BLP training strongly enhanced both phagocytosis and intracellular killing of *S. aureus* in wide‐type (WT) macrophages, these capabilities were significantly diminished in *Irg1* ^−/^ macrophages (Figure 8I). These findings collectively indicate that the potent anti‐inflammatory and anti‐infectious effects observed in BLP‐trained macrophages are predominantly dependent on the IRG1‐itaconate axis.

### 2.7 Translational Attempts to Attenuate Sepsis Lethality by Adoptive Transfer of BLP‐Trained Macrophages

**Figure 9:**
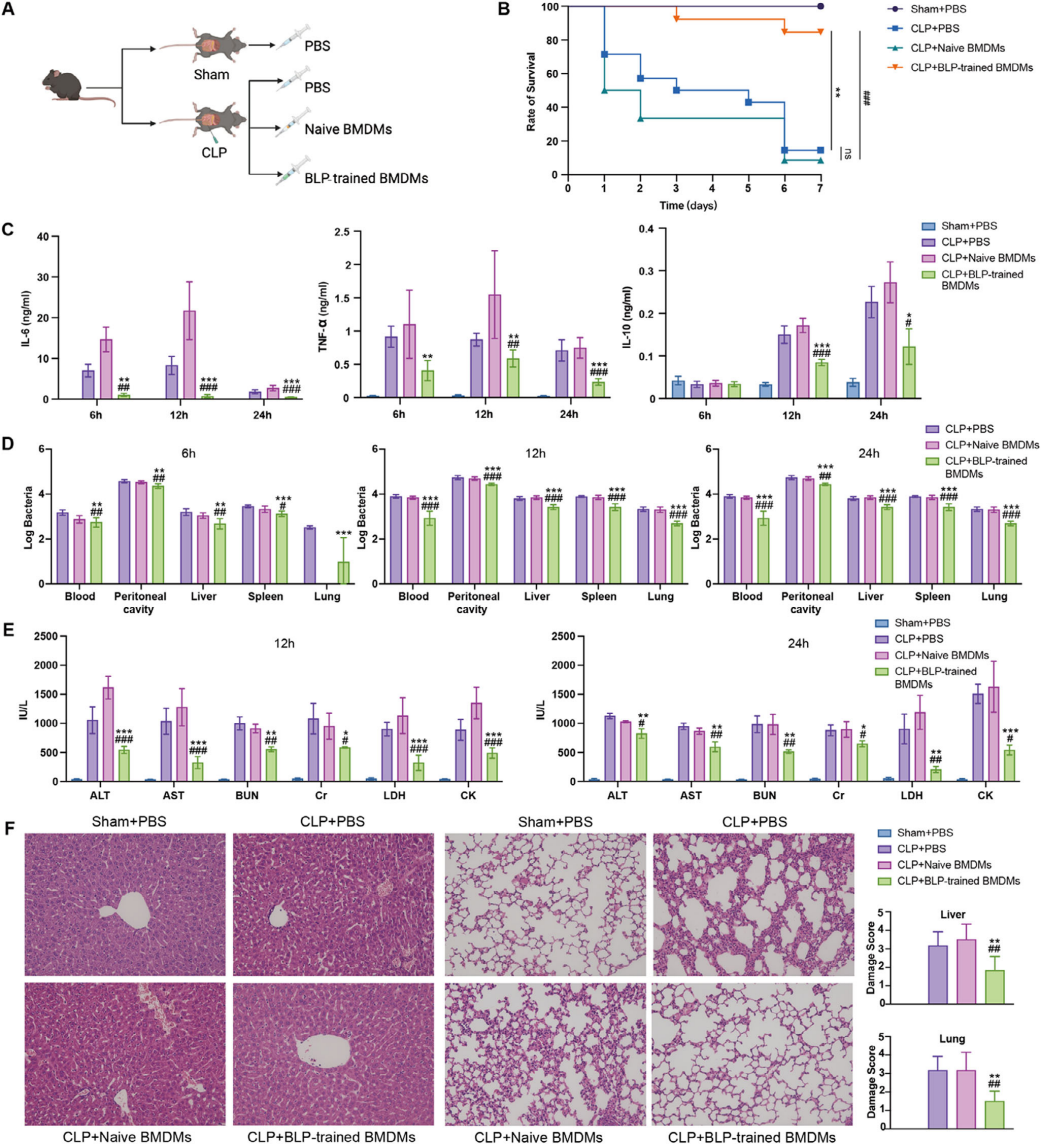
Adoptive transfer of BLP‐trained BMDMs protects mice against CLP‐induced microbial sepsis. A) Mice were challenged with cecal ligation and puncture‐induced polymicrobial sepsis and immediately injected with PBS, Naive BMDMs, or BLP‐trained BMDMs. Mice subjected to sham‐CLP were used as Sham (n = 5). B) Compared with PBS‐treated mice (n = 13) and mice receiving adoptive transfer of naive BMDMs (n = 14), mice receiving adoptive transfer of BLP‐trained BMDMs (n = 13) displayed a significantly higher survival rate (p = 0.001) upon CLP‐induced severe polymicrobial sepsis. C) Data shown are the results of plasma IL‐6, TNF‐α, and IL‐10 levels at 6, 12, and 24 h post septic challenge (n = 6–8 mice each group). D) Bacterial counts in blood, peritoneal cavity, liver, spleen, and lungs assessed at 6, 12, and 24 h post‐septic challenge (n = 6–8 mice each group). E) Serum ALT, AST, BUN, Cr, LDH, and CK levels were assessed at 12 and 24 h post‐CLP to reflect sepsis‐induced tissue and organ injury (n = 6–8 mice each group). F) Eosin‐hematoxylin staining was evaluated at 12 h post‐CLP to reflect sepsis‐induced liver and lung injury (n = 6–8 mice each group). Data in (C–E) are mean ± SD (4–6 mice per time point) and representative of three independent experiments. ^*^ *p< 0.05*, ^**^ *p< 0.01*, ^***^ *p< 0.001* vs PBS‐treated mice; ^#^ *p< 0.05*, ^##^ *p< 0.01*, ^###^ *p< 0.001* vs Naive BMDMs adoptive transfer mice.

The above in vitro work has shown that BLP‐trained BMDMs, in sharp contrast to naïve BMDMs, display strong anti‐inflammatory and anti‐infection capabilities; however, it is unclear whether in vivo adoptive transfer of BLP‐trained BMDMs affords protection against sepsis‐associated lethality. To test this, we injected BLP‐trained BMDMs into the septic mice through the tail vein immediately after the induction of CLP‐induced polymicrobial sepsis, and meanwhile, CLP‐challenged mice received either PBS or naive BMDMs intravenously used as the control (**Figure** **9**A). Adoptive transfer of BLP‐trained BMDMs resulted in a significantly improved survival from 15.3% seen in CLP‐challenged mice to 84.6% (*p* = 0.0004), whereas adoptive transfer of naïve BMDMs failed to rescue the septic mice (*p* = 0.2788) (Figure 9B). Notably, mice that received adoptive transfer of naive BMDMs exhibited an even higher mortality rate at 15 h after CLP compared to the PBS‐treated, CLP‐challenged mice (Figure S9, Supporting Information).

We next assessed the impact of adoptive transfer of BLP‐trained BMDMs on host resistance to bacterial infection as represented by the systemic inflammatory response, bacterial clearance, and tissue and organ injury. CLP‐induced sepsis caused an early elevation in circulating proinflammatory cytokines TNF‐α and IL‐6 as well as a later elevation in circulating anti‐inflammatory cytokine IL‐10 (Figure 9C), whereas adoptive transfer of BLP‐trained BMDMs substantially attenuated sepsis‐induced elevation in both proinflammatory and anti‐inflammatory cytokines (*p*< 0.05 to *p*< 0.001 vs PBS‐treated, CLP‐challenged mice) (Figure 9C). In consistent with an elevated early mortality rate, mice that received adoptive transfer of naive BMDMs showed higher circulating proinflammatory cytokines IL‐6 and TNF‐α at 6 and 12 h after CLP compared to the PBS‐treated, CLP‐challenged mice. Notably, CLP‐challenged mice showed enhanced bacterial accumulation in the circulation, peritoneal cavity, and visceral organs; however, adoptive transfer of BLP‐trained BMDMs significantly diminished bacterial counts in the circulation, peritoneal cavity, and visceral organs, including the liver, spleen, and lungs at 6, 12, and 24 h after CLP challenge (*p*< 0.05, *p*< 0.01 vs PBS‐treated, CLP‐challenged mice) (Figure 9D), indicating that adoptive transfer of BLP‐trained BMDMs accelerates bacterial clearance both locally and systemically. By contrast, adoptive transfer of naïve BMDMs was unable to reduce bacterial burden in the circulation, peritoneal cavity, and visceral organs (Figure 9D). We further evaluated the protective effect of adoptive transfer of BLP‐trained BMDMs on tissue and organ injury in CLP‐challenged mice by selecting serum lactate dehydrogenase (LDH) as an indicator for lung and general cellular damage; creatine kinase (CK) as an indicator for muscle, heart, and brain damage; alanine aminotransferase (ALT) and aspartate aminotransferase (AST) as indicators for liver damage; and blood urea nitrogen (BUN) and creatinine (Cr) as indicators for kidney damage. These parameters were measured at 12 and 24 h after CLP challenge to reflect injuries to these tissues and organs. Adoptive transfer of BLP‐trained BMDMs strongly attenuated CLP‐induced elevations in serum levels of ALT, AST, BUN, Cr, LDH, and CK at 12 and 24 h post‐CLP (*p*< 0.05 to *p*< 0.001 vs PBS‐treated, CLP‐challenged mice) (Figure 9E), indicating alleviated tissue and organ injury. In consistency with the above findings, hematoxylin‐eosin staining revealed that adoptive transfer of BLP‐trained BMDMs substantially mitigated liver and lung damage in CLP‐challenged mice in comparison with PBS‐treated or naïve BMDMs‐transferred, CLP‐challenged mice (Figure 9F). These results indicate that BLP‐trained BMDMs afforded protection against sepsis‐induced lethality is closely associated with their strong capabilities in attenuating systemic inflammatory response, accelerating bacterial clearance, and alleviating tissue and organ injury.

## 3 Discussion

Macrophages, the main phagocytes of innate immunity, are characterized by strong heterogeneity and plasticity,^[^ ^51^ ^]^ and these cells can alter their phenotypes in response to changes in the microenvironment and exhibit unique functions to maintain the homeostasis of the local microenvironment.^[^ ^52^ ^]^ Based on the different activation status and functions, macrophages are classified into classically activated macrophages (M1), which orchestrate positive immune responses and immune surveillance by secreting proinflammatory cytokines and chemokines, and alternatively activated macrophages (M2), which downregulate immune responses by secreting anti‐inflammatory cytokines IL‐10 and TGF‐β, thereby playing an important role in immune regulation.^[^ ^53^ ^]^ However, accumulated evidence has revealed that macrophage differentiation is an intricately multidimensional and dynamic process, rendering the M1/M2 classification of macrophages overly simplistic and insufficient to encapsulate the full extent of their heterogeneity.^[^ ^54^, ^55^ ^]^ Therefore, there is an urgent need for advanced new technologies and methods to discern and characterize various macrophage phenotypes and their heterogeneity, thereby deepening our understanding of how macrophages respond to infection and combat various pathogenic microorganisms. In the present study, we employed single‐cell transcriptomics to analyze the subpopulation changes and phenotypic shift of naïve and BLP‐trained mouse BMDMs in the resting status and after bacterial infection. We found that macrophages were divided into 13 subpopulations, of which six subtypes, namely C2, C4, C8, C9, C10, and C11, exhibited unique functional characteristics in the resting status, while two new subtypes emerged following BLP training, namely Mta (C5) and Mtb (C7). Consistent with our findings, Lee et al. utilized scRNA‐seq to analyze murine macrophages subjected to LPS stimulation at a subclinical dose for 5 days and identified a total of 11 subpopulations, of which naïve macrophages were mainly distributed in 7 clusters, while most LPS‐stimulated macrophages were clustered in two subtypes, namely ML1 (C4) and ML2 (C5).^[^ ^56^ ^]^ This similarity suggests that macrophages are composed of multiple cell subpopulations with distinct functions in their resting status, and that training with PAMP molecules BLP and LPS can induce the differentiation of new cell subpopulations.

Although both BLP training and LPS training can induce new cell subpopulations, substantial differences exist in DEGs and functional characteristics between BLP training‐ and LPS training‐induced subpopulations. Lee et al. found that LPS training‐induced ML1 (C4) and ML2 (C5) highly expressed chemokines *Ccl2*, *Ccl6*, *Ccl9*, and *Cxcl16*; however, ML1 (C4) cells preferentially expressed C5ar1 and Ccr5, resembling intermediate inflammatory monocytes, while ML2 (C5) cells preferentially express Ccr2, Cx3cr1, and Il18, similar to classical Ly6Chi inflammatory monocytes.^[^ ^56^ ^]^ In the present study, we found that BLP training resulted in the differentiation of two unique cell subpopulations, Mta (C5) and Mtb (C7). The upregulated genes in Mta (C5) included *Clec4e*, *Slc7a11*, *Prdx6*, *Prdx1*, *Cxcl2*, etc., which are mainly related to anti‐oxidative stress‐associated damage, hydrogen peroxide metabolism, glutathione metabolism, and anti‐inflammatory pathways, while the upregulated genes in Mtb (C7) included *Saa3*, *Il1b*, *Macro*, *Adgre1*, *Lcn2*, etc., which are linked to activation of immune‐related signaling pathways, leukocyte chemotaxis and migration, enhancement of antibacterial defense capabilities, and regulation of cytokine and chemokine production and secretion. These results indicate that there are significant differences in the specifically expressed genes and functional characteristics between the newly induced cell subpopulations by BLP training and LPS training.

It is worth noting that in the present study, we also found some genes, such as *Slc25A37*, *A2aR*, and *Elk3*, that behave as NT genes in LPS‐trained macrophages, act as T genes in BLP‐trained macrophages. SLC25A37 is a solute carrier in the inner mitochondrial membrane, and research has found that the PINK1‐PARK2 pathway‐mediated increase in Slc25A37 leads to mitochondrial iron accumulation, which correlates positively with the diagnosis and severity of sepsis.^[^ ^57^ ^]^ A2aR is a receptor for extracellular adenosine‐triggered immunosuppressive signaling, and consequently, A2aR‐deficient mice display an increased resistance to post‐septic infection.^[^ ^58^ ^]^ Elk‐3 is a member of the ETS protein family, which is pivotal in immune responses, and acts as a transcriptional repressor to regulate HO‐1 expression.^[^ ^59^ ^]^ Consequently, overexpression of Elk‐3 led to a reduction in macrophage‐mediated phagocytosis, consistent with the diminished phagocytic activity observed in LPS‐trained macrophages.^[^ ^60^ ^]^ These findings not only provide deeper insights into the differential mechanisms at play between BLP training and LPS training but also suggest a possible explanation for their distinct outcomes, as the switch of certain genes like *Slc25A37*, *A2aR*, and *Elk3* from the NT to T status could be a critical factor in avoiding late immune paralysis often associated with LPS training and affording protection against sepsis seen in BLP training. We noticed that the main feature in Mta (C5) was the enhanced resistance to the oxidative stress response, a previously unreported characteristic. It has been shown that upon bacterial infection, macrophages, on the one hand, need to generate a large amount of ROS to kill the invaded bacteria, while on the other hand, excessive ROS may cause cellular oxidative damage and ultimately cell death.^[^ ^61^ ^]^ Therefore, augmenting cellular capability to withstand the oxidative stress response is vital for maintaining cellular homeostasis in the face of bacterial infections.^[^ ^62^ ^]^

Ferroptosis is a newly discovered cell death form, which is most closely linked to oxidative damage.^[^ ^63^ ^]^ It was reported that a surge of ferrous iron levels in macrophages occurred within 1–6 h after *S. aureus* infection and subsequently initiated cell death,^[^ ^64^ ^]^ whereas inhibiting ferroptosis substantially mitigated *S aureus*‐triggered cell death, thereby enhancing the bactericidal activity of macrophages.^[^ ^65^ ^]^ Thus, we next examined the influence of BLP training on both the oxidative stress response and ferroptosis. Studies have shown that the underlying mechanism(s) of ferroptosis are mainly related to the depletion of glutathione (GSH), inactivation of glutathione peroxidase 4 (GPX4), accumulation of intracellular iron, and lipid peroxidation in the cell membrane.^[^ ^66^ ^]^ The anti‐ferroptosis function of GSH is predominantly dependent on GPX4, as GSH directly reduces toxic lipid hydroperoxide (PL‐OOH) to non‐toxic phospholipid alcohol (PL‐OH) by converting GSH into oxidized GSH, thereby attenuating lipid peroxidation.^[^ ^65^, ^67^ ^]^ In this way, inhibition of either GSH synthesis or utilization triggers the occurrence of ferroptosis.^[^ ^68^ ^]^ We analyzed DEGs following BLP training and found that BLP‐trained macrophages exhibited increased expression of *Slc3a2*, *Slc7a11*, *Gss*, and *Gclm*, enzymes linked to GSH production, reduced expression of *Steap3* and *Ncoa4*, enzymes responsible for converting Fe^3+^ to free Fe^2+^, and upregulated expression of FTL, which stabilizes the storage of iron ion. Thus, we speculated that BLP training could inhibit the Fenton reaction triggered by free iron ions on the one hand, and enhance GSH synthesis on the other hand, thereby increasing GPX4 activity and attenuating ferroptosis. This speculation was further supported by our results, as evidenced by elevated levels of proteins or enzymes involved in GSH metabolism, increased GSH levels, and reduced iron ion accumulation and intracellular ROS levels in BLP‐trained macrophages. In addition to regulation of GSH production, we also found that BLP‐trained macrophages augmented their antioxidative capability by upregulating several key antioxidant molecules such as SOD2, CAT, GSTA3, and PRDX6^[^ ^69^ ^]^ In particular, the transcription factor NRF2 pathway, which plays an important regulatory role in resisting the oxidative stress response,^[^ ^40^ ^]^ was significantly more activated in BLP‐trained macrophages than in naïve macrophages, while knocking out NRF2, the ability of BLP‐trained macrophages to resist ferroptosis upon bacterial infection was markedly impaired. These findings suggest that the NRF2 signaling pathway plays a central role in BLP training‐induced enhancement of antioxidative stress response and stabilization of iron metabolism, thereby augmenting the resistance of macrophages to oxidative stress‐associated damage and even cell death during bacterial infection.

Activation of NRF2 affects macrophage intermediary metabolism, promotes mitochondrial fusion, and regulates the expression of key metabolic enzymes and cytokines,^[^ ^70^, ^71^ ^]^ and more importantly, NRF2 activation‐triggered metabolic shifts, such as the switch from oxidative phosphorylation to glycolysis, are similar to the cellular reprogramming observed in trained innate immune responses. Furthermore, studies have shown that NRF2 activation inhibits the production of type I interferons and downregulates proinflammatory cytokines such as IL‐6 and IL‐1β, an effect independent of its classical antioxidant function.^[^ ^72^, ^73^ ^]^ Although the current research work has not yet directly established NRF2 as a central component of trained immunity, the well‐documented influence of NRF2 activation on metabolic reprogramming and cytokine production, core features of trained immune responses, positions it as a potential upstream modulator of this process. Therefore, while the definitive molecular pathways are yet to be fully elucidated, the regulatory effect of NRF2 on macrophage plasticity warrants further investigation; even if it is not an initiator of the trained immune phenotype, it may still act as a potential regulator.

Although the present study did not conduct direct parallel experiments to compare BLP‐ and LPS‐induced trained immunity, integrated analysis of transcriptomic datasets has revealed that in comparison with LPS‐trained macrophages, BLP‐trained macrophages predominantly display superior antioxidant defenses and ferroptosis resistance, characterized by significant upregulation of core antioxidant genes, including *Sod2*, *Prdx6*, *Hmox1*, etc., following BLP training and sustained elevation of antioxidant genes coupled with the suppression of pro‐ferroptosis genes, including *Nos2*, *Trf*, etc., upon secondary stimulation. These findings suggest that BLP training may enhance cellular stress tolerance through a unique epigenetic reprogramming mechanism. Future studies are needed to directly compare the effects of different interventions, namely BLP training and LPS training, on epigenetic regulatory networks through rigorous parallel control experiments to further validate the characteristic regulatory mechanisms of BLP training.

Cellular metabolism is a key factor in determining cell physiological functions, and innate immune cells respond to environmental changes such as bacterial infection through metabolic reprogramming. Although the main function of the metabolic process of the cell is to generate essential energy in the form of ATP, metabolites generated from cellular metabolism play an important role in signal transduction and regulation of immune responses. Emerging evidence has shown that immune training is closely related to the reprogramming of cellular metabolism. To understand the metabolic status of BLP‐trained macrophages, we first analyzed single‐cell transcriptome data and verified it by Western blot and metabolomic analyses. We found that BLP‐trained macrophages exhibited strong glycolysis as evidenced by upregulated *Slc2a1*, *Hk2*, *Ldha*, and increased metabolites pyruvate, lactate, and TCA cycle as evidenced by upregulated *Aco2*, *Ogdh*, *Suclg1*, *Sdhb*, *Fh1*, *Mdh2*, and increased metabolites citric acid, succinic acid, malic acid, and oxaloacetate. Notably, several anti‐inflammatory substances, including itaconic acid, citraconic acid, 2‐hydroxyglutarate, taurine, and antioxidant metabolites such as itaconic acid, GSH, also markedly increased. Itaconic acid, a metabolite of the TCA cycle converted from aconitate decarboxylation by immune response gene 1 (IRG1)‐encoded aconitate decarboxylase 1 (ACOD1), functions as an anti‐inflammatory molecule and has received the most attention in recent years.^[^ ^74^ ^]^ The results in the present study indicate that the potent anti‐inflammatory and anti‐infectious effects observed in BLP‐trained macrophages are predominantly dependent on the IRG1‐itaconate axis. Studies have found that the IRG1‐itaconate pathway in macrophages upon LPS stimulation or bacterial infection is significantly activated, and increased itaconate may exert anti‐infectious effects through its antibacterial, antioxidant, and anti‐inflammatory effects.^[^ ^70^, ^75^, ^76^, ^77^, ^78^, ^79^, ^80^ ^]^ Current research believes that the anti‐inflammatory effect of itaconic acid is mediated mainly through the following two pathways: 1) by inhibiting Keap1 and facilitating the nuclear translocation of NRF2 to promote the antioxidative response,^[^ ^70^ ^]^ 2) by suppressing *Ikbz* and GSDMD via activation of ATF3 to inhibit the proinflammatory response.^[^ ^81^ ^]^ Citraconic acid, an isomer of itaconic acid, has been recently identified as a potent activator of NRF2.^[^ ^82^ ^]^ Taurine, on the other hand, may contribute to cellular protection by inhibiting mitochondrial dysfunction, reducing DNA damage, and attenuating the inflammatory response.^[^ ^83^ ^]^ Similarly, 2‐hydroxyglutarate has emerged as a novel anti‐inflammatory metabolite.^[^ ^84^ ^]^ However, whether these metabolites are related to the pronounced anti‐inflammatory, antibacterial, and antioxidant stress properties of BLP‐trained macrophages needs further investigation.

Previous studies have shown that the most prominent hallmark in BLP‐trained macrophages is a substantially attenuated inflammatory response to a second stimulus, such as bacterial infection, and simultaneously, a significantly enhanced capability to eradicate the invaded bacteria. The present study not only supports these earlier observations but also has new findings. First, we identified that the anti‐inflammatory effect of BLP training, in addition to previously demonstrated downregulation of both NF‐κB and MAPK inflammatory signaling pathways,^[^ ^85^ ^]^ was closely associated with a substantial number of NT genes induced by BLP training, which exhibit anti‐inflammatory properties, such as *Saa3*, *Grx1*, and *Egr1*. Saa3, an acute‐phase protein, is predominantly involved in the regulation of inflammatory responses, and mice deficient in *Saa3* display a more severe inflammatory response and an impaired neutrophil bactericidal function.^[^ ^86^ ^]^ Grx1, a member of the oxidoreductase family and a component of the endogenous antioxidant defense system, has been shown to exert anti‐inflammatory effects by inhibiting the activation of MAPK and NF‐κB pathways in RAW264.7 macrophages.^[^ ^87^ ^]^ EGR1, a transcription factor involved in tissue damage and immune response regulation, has been found to suppress inflammation through its interaction with the NuRD core repressor complex.^[^ ^39^ ^]^ Second, we observed an upregulation of molecules related to phagocytosis and bacterial killing, such as *Marco*, *Fpr1*, and *Sdc1*. Marco is a scavenger receptor on macrophages and mediates opsonin‐independent phagocytosis. It has been shown that reduced Marco expression impairs phagocytosis of macrophages but does not affect LPS tolerance‐induced suppression of inflammation.^[^ ^88^ ^]^ Fpr1 is a pattern recognition receptor, and its activation significantly boosts macrophage‐associated phagocytosis of microbial pathogens such as *S. aureus* through the upregulation of receptors CR1, FCyRI, and CR3.^[^ ^89^ ^]^ Sdc1 (CD138) is a member of the cell surface proteoglycan family, and it has been found that CD138‐positive macrophages display an anti‐inflammatory phenotype and robust phagocytic ability, thereby promoting the resolution of infection.^[^ ^90^ ^]^ Simultaneously, these cells show strong proliferation characteristics and viability.^[^ ^91^ ^]^ Third, we observed that a substantially augmented antioxidative stress response in BLP‐trained macrophages is beneficial for protecting the cell from oxidative damage, maintaining cellular homeostasis, and minimizing cell death during the anti‐infection process. Finally, we found that BLP training initiated metabolic reprogramming in macrophages, thereby resulting in both an antibacterial and anti‐inflammatory effect by increasing several metabolites, in particular, itaconic acid and citraconic acid.

Mounting evidence has shown that BLP‐trained macrophages, as a special group of “well‐trained” phagocytes, are characterized by augmented bactericidal activity and attenuated proinflammatory responses, thereby conferring protection against microbial sepsis most likely via both the control of early bacterial infection and prevention of cytokine storm.^[^ ^5^ ^]^ As expected, in the present study, we found that adoptive transfer of BLP‐trained BMDMs protected CLP‐challenged mice against sepsis‐associated lethality with a significantly improved survival rate. By contrast, adoptive transfer of naïve BMDMs failed to protect septic mice, and the early mortality rate in mice that received naïve BMDMs was even higher than that in mice that received PBS at 15 h post CLP. Furthermore, serum levels of proinflammatory cytokines IL‐6 and TNF‐α in the CLP+Naive BMDMs group were markedly higher than those in the CLP+PBS group. These results imply that adoptive transfer of naive BMDMs may trigger a hyperinflammatory response and promote the onset of systemic inflammatory response syndrome (SIRS), thereby failing to confer an overall survival benefit. Consistent with our findings, other research work has also reported that adoptive transfer of naive macrophages was unable to improve survival in *S. aureus* sepsis models–despite an enhanced bacterial clearance, it triggered excessive release of proinflammatory cytokines, thereby exacerbating SIRS.^[^ ^92^ ^]^

Taken together, in the present study, we discovered 13 macrophage subpopulations and revealed a series of statuses of macrophages at rest and during activation, which are more complex than the conventional M1 and M2 polarization statuses. Importantly, BLP training resulted in the generation of two unique macrophage subpopulations, namely C5 and C7, and these two subtypes displayed significantly increased anti‐inflammatory and antibacterial‐related gene expression as well as substantially enhanced antioxidative stress capabilities. Notably, BLP‐trained macrophages exhibited an augmented antioxidative stress response via activation of the NRF2 signaling pathway, thereby mitigating oxidative stress‐induced cell damage and ferroptosis. Finally, BLP‐trained macrophages showed strong abilities in glycolysis and the TCA cycle, together with increased substances and metabolites with anti‐inflammatory and antioxidative properties. These findings provide a new perspective for our in‐depth understanding of the heterogeneity in macrophages as well as the characteristics and functional changes of macrophage subpopulations in their immune responses to bacterial infection, which are of importance for further elucidating the underlying mechanism(s) by which BLP training affords protection against microbial infection and sepsis.

## 4 Experimental Section

### Reagents and Antibodies

The TLR2 agonist BLP, a synthetic bacterial lipopeptide (Pam3 Cys‐Ser‐Lys4‐OH), was purchased from EMC Microcollections (Tubingen, Germany). The ReverTra Ace qPCR RT Kit and SYBR Green QPCR Master Mix were purchased from TOYOBO (Osaka, Japan). Antibodies that specifically target GPX4, FTL, GSS, HMOX1, GCLM, NRF2, KEAP1, SOD2, PRDX6, SLC7A11, GSTA3, GCLC, CAT, CFP, MARCO were purchased from proteintech (Wuhan, China). Antibodies that specifically target ACOD1 were purchased from Abcam (Cambridge, United Kingdom). The Reactive Oxygen Species Assay Kit was purchased from Beyotime Biotechnology (Shanghai, China). FerroOrange (Fe^2+^ indicator) was purchased from GlpBio (Montclair, California, USA). PE anti‐mouse F4/80 antibody was purchased from BioLegend (San Diego, California, USA).

### Macrophage Isolation and Cultures

Mice were maintained and used in accordance with the National Institutes of Health Guidelines for the Care and Use of Laboratory Animals. C57BL/6 mice, *Nrf2* ^−/−^ mice (B6.129×1‐Nfe212tm1Ywk/J, Jackson Laboratory, stock no. 017009), and wild‐type (WT) littermates (8 weeks old, 18–22 g) were housed under specific pathogen‐free conditions at the Animal Experimental Centre of Southern Medical University, Guangzhou, China. Environmental conditions included a temperature of 23–25 °C, humidity of 50 ± 5%, and a 12 h light/dark cycle, with ad libitum access to food and water. After genotyping, mice were acclimatized for one week prior to experiments. BMDMs were isolated and cultured as previously described.^[^ ^93^ ^]^ Briefly, bone marrow cells were flushed from femurs and tibiae with DMEM, resuspended in DMEM supplemented with 20% L929‐conditioned medium, 20% fetal bovine serum (FBS), 100 U mL^−1^ penicillin, and 100 µg mL^−1^ streptomycin, and plated in culture dishes. After 7 days of incubation, adherent cells were harvested using 0.25% trypsin (Gibco, USA). Macrophage purity exceeded 95%, as determined by flow cytometric analysis of F4/80 antigen staining using a rat anti‐mouse F4/80 antibody.

### Induction of BLP‐Trained Tolerance

To establish BLP‐trained tolerance, BMDMs were isolated and incubated with either culture medium alone as a control (Naive) or with 100 ng mL^−1^ of BLP (BLP‐trained) for 24 h prior to the bacterial challenge, following previously described methods.^[^ ^3^ ^]^

### Bacteria and Bacterial Culture

Gram‐positive *S. aureus* was obtained from the Department of Microbiology, School of Public Health, Southern Medical University, China. *S. aureus* was cultured in Luria‐Bertani broth at 37°C with agitation and was then harvested at the mid‐logarithmic growth phase, washed twice, and resuspended in Dulbecco's Phosphate Buffered Saline (DPBS) for in vitro use. The bacterial inoculum was subsequently prepared by pelleting at 5000×g in a microfuge and resuspension in DPBS. Bacterial counts were calculated by measuring the optical density using a spectrophotometer (A600).

### Single‐Cell RNA Isolation and Sequencing

BMDMs were infected with *S. aureus* at a ratio (cell: bacteria = 1:30) for the indicated time periods at 37°C. Then, the cells were washed with DPBS three times and suspended with recombinant trypsin (Gibco, Invitrogen, USA) to collect the cells. The cells were resuspended with PBS containing 0.04% BSA and subjected to single‐cell sequencing. Due to excessive cell death in the control group 6 h after bacterial infection, resulting in cell viability falling below the threshold required for single‐cell RNA sequencing (>75%), seven sequencing samples were ultimately obtained.

RNA was measured using a Bioanalyzer 2100 (Agilent Technologies). Chromium single‐cell libraries were prepared according to the standard protocol outlined in the manual. Libraries were then sequenced as 150 bp paired‐end reads on an Illumina HiSeq Xten. Read 1 contains cell barcodes and UMI, while Read 2 contains corresponding RNA sequences.

### Preprocessing of Single‐Cell RNA‐Seq Data

The fastq files from the sequencer were generated using the “mkfastq” module of the 10X Genomics Cellranger software (version 6.0). Following the generation of fastq files, the “count” module of Cellranger was employed to align the raw sequence reads to the mouse reference genome (mm10). The “count” module created three data files (barcodes.tsv, genes.tsv, matrix.mtx), which were loaded into the R package Seurat (version 3.2.0).^[^ ^94^ ^]^ The Seurat vignette (https://satijalab.org/seurat/pbmc3k_tutorial.html) was followed to create the Seurat data matrix object. In brief, all genes expressed in more than three cells and cells with at least 200 detected genes were kept. Cells with unique gene counts > 6000 or < 200 were discarded. The data were normalized using Seurat's “NormalizeData” function, which uses the “LogNormalize” method to normalize the expression of each gene for each cell to the total gene expression in that cell. The result is multiplied by a scale factor of 1e4, and the result is log‐transformed.

For integration, the top 2000 highly variable genes were identified using Seurat's *FindVariableFeatures* function. Dimensional reduction during anchor finding was performed using canonical correlation analysis (CCA), as implemented in Seurat's *FindIntegrationAnchors* function. Integration anchors were identified across datasets split by the “treatment” condition (naïve vs. treated), and batch effects were corrected using Seurat's *IntegrateData* function. Then the variation arising from library size was regressed out using the function “ScaleData” in Seurat. PCA of the variable genes was performed as input and determined significant principal components on the basis of the “JackStraw” function in Seurat. The first 15 principal components were selected as input for tSNE using the functions “FindClusters” and “RunTSNE” in Seurat. To identify differentially expressed genes (DEGs) in each cell cluster, the function “FindAllMarkers” in Seurat was used on the normalized gene expression data.

### Gene Enrichment Analysis and Cell‐Specific Gene Set Scoring Analysis

In this study, the enrichment of GO terms within differentially expressed genes (DEGs) was investigated using the ‘enrich GO’ function available in the R package clusterProfiler, as outlined by Wu et al.^[^ ^95^ ^]^ For the KEGG pathway enrichment analysis, the online resource DAVID (Database for Annotation, Visualization, and Integrated Discovery) accessible at https://david.ncifcrf.gov/summary.jsp was utilized. Gene Set Enrichment Analysis (GSEA) was conducted using the GSEA_4.3.2 software. Additionally, transcription factor enrichment analysis was performed using TRRUST (version 2), which is accessible online at https://www.grnpedia.org/trrust/. *p*‐values were adjusted for multiple testing by estimating the false discovery rate (FDR). Cell‐specific gene set scoring was analyzed for naive and BLP‐trained macrophages using the KEGG collection in the Molecular Signatures Database (MSigDB) (category, C2; subcategory, CP: KEGG)^[^ ^96^ ^]^ according to the vignette in the R package VAM (version 0.4.0, http://www.dartmouth.edu/~hrfrost/VAM/VAM_PBMC3K_ SCTransform.pdf).

### Developmental Trajectory Inference

Pseudo‐time analysis was performed on the separated Seurat object containing either naïve or trained macrophages. Each dataset was individually analyzed using the R package “VECTOR”.^[^ ^97^ ^]^ Outputs were obtained detailing the pseudo‐time cell distributions for each cell type.

### Analysis of T and NT Genes

Following the pipeline in Foster, S. L's paper,^[^ ^21^ ^]^ genes with an average normalized expression level below 0.1 were excluded across all cells first. Then, Class T genes are defined as those exhibiting an increase of over 25% in naive macrophages stimulated by *S. aureus*, alongside a decrease of more than 25% in tolerant macrophages also stimulated by *S. aureus* (i.e., N0/N3< 0.75 and T3/N3< 0.75). Conversely, Class NT genes are identified as those that not only are induced to rise by more than 25% in *S. aureus*‐stimulated naive macrophages but also maintain or exceed this elevated expression in BLP‐trained macrophages under the same *S. aureus* stimulation (that is, N0/N3< 0.75 and T3/N3 ≥ 1). To enhance clarity and facilitate interpretation, all gene expression values at N3 have been standardized to 1, and furthermore, all values have been transformed using the log10 scale. The terminology includes “N0” for naive BMDMs, “N3” for naive BMDMs stimulated with *S. aureus* for 3 h, and “T3” for BLP‐trained BMDMs stimulated with *S. aureus* for 3 h.

### Real‐Time Quantitative RT‐PCR Analysis

Naive and BLP‐trained BMDMs were incubated with *S. aureus* at a cell‐to‐bacteria ratio of 1:30 and maintained at 37°C for the indicated time periods. Subsequently, the cells were collected and lysed. Total RNA was then extracted from the BMDMs using TRIzol reagent (Invitrogen, USA) and reverse transcribed using a ReverTra Ace qPCR RT kit, adhering to the manufacturer's guidelines. RT‐qPCR was performed utilizing the SYBR Green QPCR master mix in conjunction with a 7500 real‐time PCR system (Applied Biosystems, USA). The primer sequences for RT‐qPCR are listed in the Supporting Information.

### Western Blot Analysis

Naive and BLP‐trained BMDMs were exposed to *S. aureus* at a 1:30 cell‐to‐bacteria ratio and incubated at 37°C for various durations. Following incubation, the cells were collected and lysed at the indicated time periods. Proteins extracted from each sample in equal amounts were resolved using 12% SDS‐polyacrylamide gel electrophoresis and subsequently transferred onto PVDF membranes. These membranes were blocked at room temperature for 2 h using PBS supplemented with 0.05% Tween‐20 and 5% nonfat milk. The blocking was followed by an overnight probing at 4°C with primary antibodies. The blots were then incubated with anti‐mouse or anti‐rabbit secondary antibodies at room temperature for 1 h, developed using a chemiluminescent substrate, and visualized using a Kodak IS4000R imaging system (Kodak, USA). For densitometric analysis of the Western blots, ImageJ software (version 1.42, National Institutes of Health, USA) was employed.

### Immunofluorescence

In this experiment, 1 × 10^5^ BMDMs were cultured in 12 mm Petri dishes. Following stimulation with *S. aureus (*macrophage:bacteria *=* 1:30) for various time periods, the cells were fixed in 4% paraformaldehyde (PFA) and permeabilized with 0.1% Triton X‐100. For PRDX6 assessment, a PE‐conjugated anti‐mouse F4/80 antibody (provided by BioLegend, USA) was used before cell permeabilization in order to ensure the specific distribution of F4/80 on the cell membrane. Confocal images were taken after cells were stained with the anti‐PRDX6 Ab or anti‐NRF2 Ab and Alexa Fluor 488‐conjugated secondary antibody using an LSM880 confocal laser scanning microscope (Zeiss, Germany).

### Flow Cytometry and Cell Sorting

Naive and BLP‐trained BMDMs were blocked with BSA on ice for 10 min, followed by incubation with anti‐MARCO antibody [EPR24317‐33] (Abcam, UK), anti‐CLEC4E/MINCLE antibody [AT16E3] (Abcam, UK), anti‐PRDX6 antibody (Proteintech, China), and PE‐conjugated anti‐mouse DCIR4 (Clec4a1) antibody (Biolegend, USA) for 30 min at 4°C in the dark. After incubation with primary antibodies, the cells were washed twice with PBS and then incubated with appropriate fluorochrome‐conjugated secondary antibodies for 30 min at 4°C. Subsequently, the cells were washed with PBS, resuspended in 1 mL of staining buffer (PBS containing 2% fetal bovine serum), and prepared for flow cytometry analysis.

For cell sorting, BMDMs stained with PRDX6 or MARCO were resuspended in sorting buffer (PBS containing 2% fetal bovine serum and 1 mM ethylenediaminetetraacetic acid) at a concentration of 20 × 10⁶ cells mL^−1^. Cell sorting was performed using a BD FACSAria III cell sorter (BD Biosciences, USA) equipped with a 100 µm nozzle. PRDX6‐positive cells (C5 subset) and MARCO‐positive cells (C7 subset) were sorted into collection tubes containing complete DMEM medium with 20% fetal bovine serum, respectively. Post‐sort purity was confirmed by reanalysis, with a minimum acceptable purity of 95%. The sorted cells were centrifuged at 300×g for 5 min at 4°C and used directly for downstream experiments or cryopreserved for later use.

### Cellular Ferrous Iron and ROS Detection

The concentrations of cytosolic ferrous iron and reactive oxygen species (ROS) were quantified using the FerroOrange probe (GlpBio) and the Reactive Oxygen Species Assay Kit (Beyotime), respectively. Briefly, for the detection of cytosolic ferrous iron and ROS, cells were first cultured in confocal Petri dishes with coverslip bottoms. Subsequently, the cells were washed three times with Hanks Balanced Salt Solution (HBSS) and incubated with 1 µM FerroOrange or 1 nM DCFH‐DA, respectively, for 30 min at 37°C and 5% CO2. Immediately after incubation, the cells were observed using a Zeiss LSM880 confocal microscope (Zeiss, Germany). To validate the specificity of FerroOrange, a parallel set of cells was co‐incubated with both FerroOrange and Deferiprone (DFP) and subsequently examined using the same microscope.

### Metabolomic Analysis

Naive and BLP‐trained BMDMs were incubated with *S. aureus* at a cell‐to‐bacteria ratio of 1:30 for 3 h (N3 and T3) or culture medium (N0 and T0). Harvest cells, then add 80% methanol, freeze‐thaw three times in liquid nitrogen, and centrifuge at 4 ° C for 20 min. Collect the supernatant and freeze‐dry it by centrifugation. Finally, the volume of the samples in the freeze‐drying tube is normalized based on the protein content of each sample. The redissolved solution is prepared using a 1:1 ratio of acetonitrile to water. The experiment was repeated four times. A sample metabolomic analysis was performed on an ultra‐performance liquid chromatography (UPLC) system coupled with an orbitrap fusion mass spectrometer (Thermo Fisher Scientific, Waltham, USA). The metabolome separation was performed on a Thermo Hyperil Gold C18 column (100 × 2.1 mm^2^, 1.9µm). In the positive ion mode, the mobile phases consisted of A: 0.1% formic acid in H_2_O and B: 0.1% formic acid in acetonitrile (ACN). In the negative ion mode, the mobile phases consisted of A: 5 mM ammonium acetate in H_2_O, pH 9, and B: ACN. The gradient elution was set as follows: 0–1 min, 5% B, 1–12 min, 5%‐100% B, 12.1‐15 min, 5% B for both positive and negative ion modes. The flow rate was set as 0.3 mL min^−1^. The injection volume was 2 µL. The column temperature was set at 30 °C for all analyses. The mass spectrometer was operated in the full‐scan mode in the range of 70–1050 m/z under both positive and negative electrospray ionization modes. The ion transfer tube temperature and vaporizer temperature were set as 350 and 320 °C, respectively. The sheath gas and aux gas were set at 40 and 10 Arb, respectively.

For metabolite Identification, the raw mass spectrometry data were processed using Compound Discoverer 3.2 (Thermo Fisher Scientific). The workflow, including Data Normalization: Raw data were normalized to correct for batch effects and instrument variability. Database Matching: Metabolites were annotated by matching experimental MS/MS spectra against authoritative databases (mzCloud, mzVault, Masslist, and Chemspider), with Parameters set as mass tolerance ≤ 5 ppm, RT tolerance ≤ 0.2 min, and minimum spectral similarity score ≥70%.

The abundance of each metabolite was measured based on its peak area in the extracted ion chromatogram (XIC). Relative quantification was performed by comparing normalized peak areas between experimental groups. The volcano plot of the raw data is placed in the Supporting Information.

### Phagocytosis and Bactericidal Assays

BMDMs were seeded at 2 × 10⁵ cells/well in two separate 24‐well culture plates (designated as plate #1 and #2) and cultured in DMEM supplemented with 10% FBS at 37°C with 5% CO_2_ until complete adherence. Following cell attachment, both WT and *Irg1* ^−/−^ BMDMs were subjected to BLP training or left untreated as controls, with PBS washing performed thrice before treatment. Live *S. aureus* was prepared in DMEM containing 1% FBS at an MOI of 30 and co‐incubated with BMDMs for 30 min. After co‐incubation, extracellular bacteria were eliminated by washing cells three times with PBS containing 0.5 µg mL^−1^ gentamicin, followed by a brief 3 min incubation with PBS containing 50 µg mL^−1^ gentamicin. For phagocytosis assessment (plate #1), cells were immediately lysed with 500 µL of 0.1% Triton X‐100 solution. For bactericidal capacity evaluation (plate #2), cells were further cultured for 60 min in DMEM with 10% FBS before lysis. Cell lysates were serially diluted (10 fold dilutions), and 100 µL of the 10^3^ dilutions were plated on LB agar plates and incubated at 37°C for ≈16 h. Bacterial colonies were enumerated on plates containing 30–300 colonies. The phagocytic capacity was determined by the bacterial count from plate #1, while the bactericidal efficiency was calculated as the percentage reduction in bacterial load between plates #1 and #2. All experiments were performed in triplicate wells with three technical replicates for each dilution.

### Mice, Septic Models, Tail Vein Injection of BMDMs

Pyrogen‐free, male C57BL/6 mice (10–12 week old and 18–22 g) were obtained from and maintained in the animal facility of Southern Medical University. Mice were housed in barrier cages under controlled environmental conditions (12/12 h of light/dark cycle, 55 6 5% humidity, and 23°C) and had free access to standard laboratory chow and water. All animal experiments in this study were approved by the Ethical Committee of Southern Medical University (Approval No. SMUL202409022) and conducted in accordance with the National Institutes of Health Guidelines for the Care and Use of Laboratory Animals.

Mice were anesthetized by i.m. injection of 150 mL of a ketamine/xylazine admixture (150 mL of ketamine and 150 mL of xylazine made up to 1 mL with 0.9% saline). A midline laparotomy was performed when the cecum was delivered and ligated at the base, just distal to the ileocecal juncture with a 2/0 MERSILK tie. A single thorough puncture was then made distal to the ligature with a 17‐gauge needle. The cecum was returned to the peritoneal cavity, and the abdomen was closed with 6/0 PROLENE Sutures. Survival was monitored for at least 7 d.

All CLP‐challenged mice were injected via the tail vein at the same time. CLP+PBS mice were injected with 0.1 mL PBS solution, CLP+Naive BMDMs mice were injected with 0.1 mL 1 × 10^7^ cells mL^−1^ Naive BMDMs, and CLP+BLP trained BMDMs mice were injected with 0.1 mL 1 × 10^7^ cells mL^−1^ BLP trained BMDMs.

### Assessment of Bacteria Loads in the Blood, Peritoneal Cavity, and Visceral Organs

Bacterial loads were determined as described previously.^[^ ^5^ ^]^ Briefly, mice were euthanized at various time points following CLP surgery. Peritoneal lavage fluid, heparinized whole blood, and visceral organs were collected, serially diluted in sterile water containing 0.5% Triton X‐100 (Sigma–Aldrich, Missouri, USA), plated onto sheep's blood agar plates (Beaver, Suzhou, China), and incubated at 37°C for 24 h for colony‐forming unit (CFU) enumeration.

### Measurement of Cytokines in Plasma and Cell Culture Supernatant

Heparinized whole‐blood samples were collected at various time points following CLP‐induced polymicrobial sepsis and centrifuged to obtain plasma. Cell culture supernatants were harvested from in vitro experiments after the indicated treatments. Concentrations of TNF‐α, IL‐6, and IL‐10 in both plasma samples and cell culture supernatants were measured by ELISA kits (Neobioscience, Shenzhen, China) in accordance with the manufacturer's instructions. All measurements were performed in triplicate.

### Assessment of Hepatic, Heart, Liver, and Renal Functions

Whole‐blood samples were collected from Sham, CLP mice, CLP+PBS mice, CLP+Naive BMDMs mice, and CLP+BLP trained BMDMs mice at different time points post‐CLP, and the hepatic, heart, liver, and renal functions were assessed by measuring serum levels of aspartate aminotransferase (AST), alanine aminotransferase (ALT), creatine kinase (CK), lactate dehydrogenase (LDH), blood urea nitrogen (BUN), and creatinine (Cr) using the Activity Assay Kit (Solarbio, Beijing, China).

### Histological Examination

Following fixation in 4% phosphate‐buffered formaldehyde (Sigma–Aldrich, Missouri, USA), tissues underwent embedding in optimum cutting temperature cryomedium (Sakura Finetek, Alphen aan den Rijn, Netherlands) and were sectioned into 4 µm slices. Histological assessment was performed using an H&E staining kit (Abcam, Cambridge, United Kingdom) to elucidate histomorphological features.^[^ ^98^ ^]^ Tissue damage was assigned following established methodologies.^[^ ^99^, ^100^ ^]^ Tissue damage scores were based on non‐normal tissue size: 0 indicated absence, 1 denoted <25%, 2 represented 25–50%, 3 indicated 50–75%, and 4 signified 75–100% involvement.

### Statistical Analysis

The data are shown as the mean values ± SEMs throughout. Statistical analyses were performed with GraphPad Prism 9 software (GraphPad, La Jolla, CA). Differences between groups were analyzed using the nonparametric Mann–Whitney test. Survival rates in animal experiments were analyzed using the Log‐rank (Mantel‐Cox) test. Differences were considered significant when *p*< 0.05, and levels of significance were specified throughout the figure legends.

## Conflict of Interest

The authors declare no conflict of interest.

## Author Contributions

Y.W., Y.H., and X.J. contributed equally to this work and share first authorship. J.L., H.L., Y.J., and T.S. contributed to the study conception and design. Y.H., Y.W., J.L., J.X., L.L., and W.C. conceived and performed the experiments. X.J., Y.W., and M.R. designed the bioinformatic algorithms for data analysis. T.X., Y.W., and Z.L. analyzed metabolomics data. J.L. and Y.W. wrote the manuscript. All authors approved the final manuscript.

## Supporting information



Supporting Information

Supporting Information

Supporting Information

## Data Availability

The data that support the findings of this study are available from the corresponding author upon reasonable request.

## References

[1] M. Cecconi , L. Evans , M. Levy , A. Rhodes , Lancet. 2018, 392, 75. 29937192 10.1016/S0140-6736(18)30696-2

[2] G. C. O'Brien , J. H. Wang , H. P. Redmond , J. Immunol. 2005, 174, 10206. 10.4049/jimmunol.174.2.1020 15634926

[3] J. Liu , J. Xiang , X. Li , S. Blankson , S. Zhao , J. Cai , Y. Jiang , H. P Redmond , J. H. Wang , Sci. Rep. 2017, 7, 40418. 28079153 10.1038/srep40418 PMC5227741

[4] S. Zhao , D. Xi , J. Cai , W. Chen , J. Xiang , N Peng , J. Wang , Y. Jiang , Z. Mei , J. Liu , Sci China Life Sci. 2020, 63, 401. 31152389 10.1007/s11427-019-9527-3

[5] W. Chen , S. Zhao , M. Ita , Y. Li , J. Ji , Y. Jiang , H. P. Redmond , J. H. Wang , J. Liu , J. Immunol. 2020, 204, 408. 31801813 10.4049/jimmunol.1801602

[6] J. Cha , I. Lee , Exp. Mol. Med. 2020, 52, 17988. 10.1038/s12276-020-00528-0 PMC8080824 33244151

[7] C. Li , A. Menoret , C. Farragher , Z. Ouyang , C. Bonin , P. Holvoet , A. T. Vella , B. Zhou , JCI Insight. 2019, 5, 126453. 30990466 10.1172/jci.insight.126453 PMC6542613

[8] D. A. Hume , S. M. Millard , A. R. Pettit , Blood. 2023, 142, 13397. 10.1182/blood.2023020597 37595274

[9] Y. Hao , T. Stuart , M. H. Kowalski , S. Choudhary , P. Hoffman , A. Hartman , A. Srivastava , G. Molla , S. Madad , C. Fernandez‐Granda , R. Satija , Nat. Biotechnol. 2023, 42, 293. 37231261 10.1038/s41587-023-01767-y PMC10928517

[10] D. Ni , H. Zhou , P. Wang , F. Xu , C. Li , Phenomics 2023, 3, 613. 38223685 10.1007/s43657-023-00129-7 PMC10781933

[11] C.‐A. Dutertre , E. Becht , S. E. Irac , A. Khalilnezhad , V. Narang , S. Khalilnezhad , P. Y. Ng , L. L. van den Hoogen , J. Y. Leong , B. Lee , M. Chevrier , X. M. Zhang , P. J. A. Yong , G. Koh , J. Lum , S. W Howland , E. Mok , J. Chen , A. Larbi , H. K. K. Tan , T. K. H. Lim , P. Karagianni , A. G. Tzioufas , B. Malleret , J. Brody , S. Albani , J. van Roon , T. Radstake , E. W. Newell , F. Ginhoux , Immunity 2019, 51, P573. 10.1016/j.immuni.2019.08.008 31474513

[12] J. Shi , K. L. Fok , P. Dai , F. Qiao , M. Zhang , H. Liu , M. Sang , M. Ye , Y. Liu , Y. Zhou , C. Wang , F. Sun , G. Xie , H. Chen , Cell Discov. 2021, 7, 34. 34001862 10.1038/s41421-021-00260-7 PMC8129088

[13] M. H. Sieweke , J. E. Allen , Science 2013, 342, 1242974. 24264994 10.1126/science.1242974

[14] J‐D Lin , H. Nishi , J. Poles , X. Niu , C. Mccauley , K. Rahman , E. J. Brown , S. T. Yeung , N. Vozhilla , A. Weinstock , S. A. Ramsey , E. A. Fisher , P. Loke , JCI Insight 2019, 4, 124574. 30830865 10.1172/jci.insight.124574 PMC6478411

[15] C. Morse , T. Tabib , J. Sembrat , K. L. Buschur , H. T. Bittar , E. Valenzi , Y. Jiang , D. J. Kass , K. Gibson , W. Chen , A. Mora , P. V. Benos , M. Rojas , R. Lafyatis , Eur. Respir. 2019, 54, 1802441. 10.1183/13993003.02441-2018 PMC8025672 31221805

[16] B. Knoops , V. Argyropoulou , S. Becker , L. Ferte , O. Kuznetsova , Mol. Cells 2016, 39, 60. 26813661 10.14348/molcells.2016.2341 PMC4749876

[17] N. S. Harasymowicz , N. Rashidi , A. Savadipour , C.‐L. Wu , R. Tang , J. Bramley , W. Buchser , F. Guilak , FASEB J. 2021, 35, 21417. 10.1096/fj.202001970R PMC8743141 33566380

[18] I. Park , M. E. Goddard , J. E. Cole , N. Zanin , L.‐P. Lyytikäinen , T. Lehtimäki , E. Andreakos , M. Feldmann , I. Udalova , I. Drozdov , C. Monaco , Nat. Commun. 2022, 13, 215. 35017526 10.1038/s41467-021-27862-9 PMC8752790

[19] L. A. Al‐Alwan , Y. Chang , A. Mogas , A. J. Halayko , C. J. Baglole , J. G. Martin , S. Rousseau , D. H. Eidelman , Q. Hamid , J. Immunol. 2013, 191, 27311. 10.4049/jimmunol.1203421 PMC3748335 23904157

[20] M. Liu , L. Yin , W. Li , J. Hu , H. Wang , B. Ye , Y. Tang , C. Huang , J. Cell. Physiol. 2019, 234, 1873147. 10.1002/jcp.28513 PMC6618013 30953351

[21] S. L. Foster , D. C. Hargreaves , R. Medzhitov , Nature 2007, 447, 972. 17538624 10.1038/nature05836

[22] E. Platanitis , T. Decker , Front Immunol 2018, 9, 2542. 30483250 10.3389/fimmu.2018.02542 PMC6242948

[23] Z. Chen , X. Wan , Q. Hou , S. Shi , L. Wang , P. Chen , X. Zhu , C. Zeng , W. Qin , W. Zhou , Z. Liu , Cell Death Dis. 2016, 7, 2068. 10.1038/cddis.2015.300 PMC4816163 26794661

[24] S. Tang , J. Zhang , L. Zhang , Y. Zhao , L. Xiao , F. Zhang , Q. Li , Y Yang , Q. Liu , J. Xu , L. Li , Hepatol Commun 2023, 7, 0257. 10.1097/HC9.0000000000000257 PMC10503672 37708451

[25] H. Zhong , H. Lin , Q. Pang , J. Zhuang , X. Liu , X. Li , J. Liu , J. Tang , Inflamm Res 2021, 70, 193. 33474594 10.1007/s00011-021-01437-2 PMC7817350

[26] H. T. Aung , K. Schroder , S. R. Himes , K. Brion , W. Van Zuylen , A. Trieu , H. Suzuki , Y. Hayashizaki , D. A. Hume , M. J. Sweet , T. Ravasi , FASEB J. 2006, 20, 13157. 10.1096/fj.05-5360com 16816106

[27] M. Fontaine , S. Planel , E. Peronnet , F. Turrel‐Davin , V. Piriou , A. Pachot , G. Monneret , A. Lepape , F. Venet , PLoS One. 2014, 9, 100909. 10.1371/journal.pone.0100909 PMC4067416 24956170

[28] C. Petes , V. Mintsopoulos , R. L. Finnen , B. W. Banfield , K. Gee , J. Biol. Chem. 2018, 293, 1763145. 10.1074/jbc.RA118.003501 PMC6231135 30242126

[29] Y. Sun , P. Caplazi , J. Zhang , A. Mazloom , S. Kummerfeld , G. Quinones , K. Senger , J. Lesch , I. Peng , A. Sebrell , W. Luk , Y. Lu , Z. Lin , K. Barck , J. Young , M. Del Rio , S. Lehar , V. Asghari , W. Lin , S. Mariathasan , J. DeVoss , S. Misaghi , M. Balazs , T. Sai , B. Haley , P. E. Hass , M. Xu , W. Ouyang , F. Martin , W. P. Lee , et al., J. Immunol. 2014, 193, 860. 24935926 10.4049/jimmunol.1400045

[30] M. Djurec , O. Graña , A. Lee , K. Troulé , E. Espinet , L. Cabras , C. Navas , M. T. Blasco , L. Martín‐Díaz , M. Burdiel , J. Li , Z. Liu , M. Vallespinós , F. Sanchez‐Bueno , M. R. Sprick , A. Trumpp , B. Sainz , F. Al‐Shahrour , R. Rabadan , C. Guerra , M. Barbacid , Proc Natl Acad Sci U S A 2018, 115, E1147. 29351990 10.1073/pnas.1717802115 PMC5819438

[31] R. Okada , K. Yamamoto , N. Matsumoto , Biochem Biophys. Rep. 2020, 24, 100840. 33294631 10.1016/j.bbrep.2020.100840 PMC7689045

[32] Y. Chang , G. Li , Y. Zhai , L. Huang , Y. Feng , D Wang , W. Zhang , H. Hu , Front Immunol. 2020, 11, 580934. 33329553 10.3389/fimmu.2020.580934 PMC7734322

[33] H. Kojima , A. Otani , A. Oishi , Y. Makiyama , S. Nakagawa , N. Yoshimura , Blood 2011, 117, 10910. 10.1182/blood-2010-05-286963 21059898

[34] B. Zou , M. Goodwin , D. Saleem , W. Jiang , J. Tang , Y. Chu , R. S. Munford , M. Lu , Elife 2021, 10. 10.7554/eLife.70938 PMC8594946 34783310

[35] Q. Li , L. Zhou , L. Wang , S. Li , G. Xu , H. Gu , D. Li , M. Liu , L. Fang , Z. Wang , S. Han , B. Zheng , Eur. J. Immunol. 2020, 50, 525. 31954378 10.1002/eji.201948299

[36] J. Wessells , M. Baer , H. A. Young , E. Claudio , K. Brown , U. Siebenlist , P. F. Johnson , J. Biol. Chem. 2004, 279, 4999503. 10.1074/jbc.M404246200 15465827

[37] O. M. Pello , M. De Pizzol , M. Mirolo , L. Soucek , L. Zammataro , A. Amabile , A. Doni , M. Nebuloni , L. B. Swigart , G. I. Evan , A. Mantovani , M. Locati , Blood 2012, 119, 411. 22067385 10.1182/blood-2011-02-339911

[38] H. T. Hop , L. T. Arayan , T. X. N. Huy , A. W. B. Reyes , S. H. Vu , W. Min , H. J. Lee , M. H. Rhee , H. H. Chang , S. Kim , Front. Cell Infect. Microbiol. 2018, 8, 287. 30186773 10.3389/fcimb.2018.00287 PMC6110913

[39] M. Trizzino , A. Zucco , S. Deliard , F. Wang , E. Barbieri , F. Veglia , D. Gabrilovich , A. Gardini , Sci. Adv. 2021, 7, aaz8836. 10.1126/sciadv.aaz8836 PMC7806227 33523892

[40] V. Ngo , M. L. Duennwald , Antioxidants (Basel) 2022, 11, 2345. 36552553 10.3390/antiox11122345 PMC9774434

[41] J. Mages , H. Dietrich , R. Lang , Immunobiology 2007, 212, 723. 18086374 10.1016/j.imbio.2007.09.015

[42] S. K. Butcher , C. E. O'Carroll , C. A. Wells , R. J. Carmody , Front Immunol 2018, 9, 933. 29867935 10.3389/fimmu.2018.00933 PMC5960718

[43] A. M. Masyutina , P. V. Maximchik , G. Z. Chkadua , M. V. Pashenkov , Front. Immunol. 2023, 14, 1006002. 36776861 10.3389/fimmu.2023.1006002 PMC9909295

[44] D. Liu , S. Cao , Y. Zhou , Y. Xiong , J. Cell. Biochem. 2019, 120, 56. 30246452 10.1002/jcb.27547

[45] J. J. Seeley , S. Ghosh , J Leukoc Biol 2017, 101, 107. 27780875 10.1189/jlb.3MR0316-118RR

[46] A. K. Bachhawat , S. Yadav , IUBMB Life 2018, 70, 585. 29667297 10.1002/iub.1756

[47] X. Chen , C. Yu , R. Kang , D. Tang , Front Cell Dev Biol 2020, 8, 590226. 33117818 10.3389/fcell.2020.590226 PMC7575751

[48] D. G. Ryan , L. A. J. O'Neill , Annu. Rev. Immunol. 2020, 38, 289. 31986069 10.1146/annurev-immunol-081619-104850

[49] C. Diskin , T. A. J. Ryan , L. A. J. O'Neill , Immunity 2021, 54, 19. 33220233 10.1016/j.immuni.2020.09.014

[50] Z. Zaslona , L. A. J. O'Neill , Mol. Cell 2020, 78, 814. 32333837 10.1016/j.molcel.2020.04.002

[51] M. Locati , G. Curtale , A. Mantovani , Annu Rev Pathol 2020, 15, 123. 31530089 10.1146/annurev-pathmechdis-012418-012718 PMC7176483

[52] Z. Bian , Y. Gong , T. Huang , C. Z. W. Lee , L. Bian , Z. Bai , H. Shi , Y. Zeng , C. Liu , J. He , J. Zhou , X. Li , Z. Li , Y. Ni , C. Ma , L. Cui , R. Zhang , J. K. Y. Chan , L. G. Ng , Y Lan , F. Ginhoux , B. Liu , Nature 2020, 582, 571. 32499656 10.1038/s41586-020-2316-7

[53] P. J. Murray , Annu. Rev. Physiol. 2017, 79, 541. 27813830 10.1146/annurev-physiol-022516-034339

[54] C. Bleriot , S. Chakarov , F. Ginhoux , Immunity 2020, 52, 957. 32553181 10.1016/j.immuni.2020.05.014

[55] M. Nahrendorf , F. K. Swirski , Circ. Res. 2016, 119, 414. 27458196 10.1161/CIRCRESAHA.116.309194 PMC4965179

[56] J. Lee , S. Geng , S. Li , L. Li , Front Immunol 2021, 12, 627036. 33708217 10.3389/fimmu.2021.627036 PMC7940189

[57] C. Li , Y. Zhang , X. Cheng , H. Yuan , S. Zhu , J. Liu , Q. Wen , Y. Xie , J. Liu , G. Kroemer , D. J. Klionsky , M. T. Lotze , H. J. Zeh , R. Kang , D. Tang , Dev. Cell 2018, 46, 441. 30100261 10.1016/j.devcel.2018.07.012 PMC7654182

[58] D. C. Nascimento , P. R. Viacava , R. G. Ferreira , M. A. Damaceno , A. R. Piñeros , P. H. Melo , P. B. Donate , J. E. Toller‐Kawahisa , D. Zoppi , F. P. Veras , R. S. Peres , L. Menezes‐Silva , D. Caetité , A. E. R. Oliveira , Í. M. S. Castro , G. Kauffenstein , H. I. Nakaya , M. C. Borges , D. S. Zamboni , D. M. Fonseca , J. A. R. Paschoal , T. M. Cunha , V. Quesniaux , J. Linden , F. Q. Cunha , B. Ryffel , J. C. Alves‐Filho , Immunity 2021, 54, 2024. 34473957 10.1016/j.immuni.2021.08.005

[59] K. Tsoyi , A. M. Geldart , H. Christou , X. Liu , S. W. Chung , M. A. Perrella , J Leukoc Biol 2015, 97, 171. 25351511 10.1189/jlb.4A0214-087R PMC4377825

[60] X. Feng , T. Deng , Y. Zhang , S. Su , C. Wei , D. Han , Immunology 2011, 132, 287. 21039473 10.1111/j.1365-2567.2010.03364.x PMC3050451

[61] A. P West , I. E. Brodsky , C. Rahner , D. K. Woo , H. Erdjument‐Bromage , P. Tempst , M. C. Walsh , Y. Choi , G. S. Shadel , S. Ghosh , Nature 2011, 472, 476. 21525932 10.1038/nature09973 PMC3460538

[62] P. Wang , J. Geng , J. Gao , H. Zhao , J. Li , Y. Shi , B. Yang , C. Xiao , Y. Linghu , X. Sun , X. Chen , L. Hong , F. Qin , X. Li , J.‐S. Yu , H. You , Z. Yuan , D. Zhou , R. L. Johnson , L. Chen , Nat. Commun. 2019, 10, 755. 30765703 10.1038/s41467-019-08680-6 PMC6376064

[63] X. Jiang , B. R. Stockwell , M. Conrad , Nat. Rev. Mol. Cell Biol. 2021, 22, 266. 33495651 10.1038/s41580-020-00324-8 PMC8142022

[64] R. Ma , L. Fang , L. Chen , X. Wang , J. Jiang , L. Gao , Theranostics 2022, 12, 22669. 10.7150/thno.66663 PMC8899587 35265210

[65] M. Dai , W. Ouyang , Y. Yu , T. Wang , Y. Wang , M. Cen , L. Yang , Y. Han , Y. Yao , F. Xu , J. Adv. Res. 2024, 62, 143 . 37777065 10.1016/j.jare.2023.09.042 PMC11331171

[66] J. Ousingsawat , R. Schreiber , E. Gulbins , M. Kamler , K. P Kunzelmann , Cell. Physiol. Biochem. 2021, 55, 590. 34637202 10.33594/000000437

[67] I. Ingold , C. Berndt , S. Schmitt , S. Doll , G. Poschmann , K. Buday , A. Roveri , X. Peng , F. Porto Freitas , T. Seibt , L. Mehr , M. Aichler , A. Walch , D. Lamp , M. Jastroch , S. Miyamoto , W. Wurst , F. Ursini , E. S. J. Arnér , N. Fradejas‐Villar , U. Schweizer , H. Zischka , J. P. Friedmann Angeli , M. Conrad , Cell 2018, 172, P409. 10.1016/j.cell.2017.11.048 29290465

[68] W. S. Yang , R. SriRamaratnam , M. E. Welsch , K. Shimada , R. Skouta , V. S. Viswanathan , J. H. Cheah , P. A. Clemons , A. F. Shamji , C. B. Clish , L. M. Brown , A. W. Girotti , V. W. Cornish , S. L. Schreiber , B. R. Stockwell , Cell 2014, 156, 317. 24439385 10.1016/j.cell.2013.12.010 PMC4076414

[69] A. Raghunath , K. Sundarraj , R. Nagarajan , F. Arfuso , J. Bian , A. P. Kumar , G. Sethi , E. Perumal , Redox Biol. 2018, 17, 297. 29775961 10.1016/j.redox.2018.05.002 PMC6007815

[70] E. L. Mills , D. G. Ryan , H. A. Prag , D. Dikovskaya , D. Menon , Z. Zaslona , M. P. Jedrychowski , A. S. H. Costa , M. Higgins , E. Hams , J. Szpyt , M. C. Runtsch , M. S. King , J. F. McGouran , R. Fischer , B. M. Kessler , A. F. McGettrick , M. M. Hughes , R. G. Carroll , L. M. Booty , E. V. Knatko , P. J. Meakin , M. L. J. Ashford , L. K. Modis , G. Brunori , D. C. Sévin , P. G. Fallon , S. T. Caldwell , E. R. S. Kunji , E. T. Chouchani , et al., Nature 2018, 556, 113. 29590092 10.1038/nature25986 PMC6047741

[71] C. Y. Lian , B. X. Chu , W. H. Xia , Z. Y. Wang , R. F. Fan , L. Wang , J. Adv. Res. 2023, 46, 87. 37003700 10.1016/j.jare.2022.04.016 PMC10105071

[72] E. H. Kobayashi , T. Suzuki , R. Funayama , T. Nagashima , M. Hayashi , H. Sekine , N. Tanaka , T. Moriguchi , H. Motohashi , K. Nakayama , M. Yamamoto , Nat. Commun. 2016, 7, 11624. 27211851 10.1038/ncomms11624 PMC4879264

[73] S. Saha , B. Buttari , E. Panieri , E. Profumo , L. Saso , Molecules 2020, 25, 5474. 33238435 10.3390/molecules25225474 PMC7700122

[74] E. M. Palsson‐McDermott , L. A. J. O'Neill , Cell Metab. 2025, 37, 10499. 10.1016/j.cmet.2025.03.004 40169002

[75] J. D. Bailey , M. Diotallevi , T. Nicol , E. McNeill , A. Shaw , S. Chuaiphichai , A. Hale , A. Starr , M. Nandi , E. Stylianou , H. McShane , S. Davis , R. Fischer , B. M. Kessler , J. McCullagh , K. M. Channon , M. J. Crabtree , Cell Rep. 2019, 28, P218. 10.1016/j.celrep.2019.06.018 PMC6616861 31269442

[76] M. Chen , H. Sun , M. Boot , L. Shao , S.‐J. Chang , W. Wang , T. T. Lam , M. Lara‐Tejero , E. H Rego , J. E. Galán , Science 2020, 369, 450. 32703879 10.1126/science.aaz1333 PMC8020367

[77] A. Hooftman , S. Angiari , S. Hester , S. E. Corcoran , M. C. Runtsch , C. Ling , M. C. Ruzek , P. F. Slivka , A. F. McGettrick , K. Banahan , M. M. Hughes , A. D. Irvine , R. Fischer , L. A. J. O'Neill , Cell Metab. 2020, 32, P468. 10.1016/j.cmet.2020.07.016 PMC7422798 32791101

[78] E‐M Schuster , M. W. Epple , K. M. Glaser , M. Mihlan , K. Lucht , J. A. Zimmermann , A. Bremser , A. Polyzou , N. Obier , N. Cabezas‐Wallscheid , E. Trompouki , A. Ballabio , J. Vogel , J. M. Buescher , A. J. Westermann , A. S. Rambold , Nat Metab 2022, 4, 856. 35864246 10.1038/s42255-022-00605-w PMC9314259

[79] J. A. Martina , R. Puertollano , Mol. Cell 2022, 82, 27324. 10.1016/j.molcel.2022.06.009 PMC12489825 35931037

[80] C. Chen , Z. Zhang , C. Liu , P. Sun , P. Liu , X. Li , Cell Metab. 2024, 36, 498. 38181789 10.1016/j.cmet.2023.12.015

[81] M. Bambouskova , L. Gorvel , V. Lampropoulou , A. Sergushichev , E. Loginicheva , K. Johnson , D. Korenfeld , M. E. Mathyer , H. Kim , L‐H Huang , D. Duncan , H. Bregman , A. Keskin , A. Santeford , R. S. Apte , R. Sehgal , B. Johnson , G. K. Amarasinghe , M. P. Soares , T. Satoh , S. Akira , T. Hai , C. de Guzman Strong , K. Auclair , T. P. Roddy , S. A. Biller , M. Jovanovic , E. Klechevsky , K. M. Stewart , G. J. Randolph , et al., Nature 2018, 556, 501. 29670287 10.1038/s41586-018-0052-z PMC6037913

[82] F. Chen , W. A. M. Elgaher , M. Winterhoff , K. Büssow , F. H. Waqas , E. Graner , Y. Pires‐Afonso , L. Casares Perez , L. de la Vega , N. Sahini , L. Czichon , W. Zobl , T. Zillinger , M. Shehata , S. Pleschka , H. Bähre , C. Falk , A. Michelucci , S. Schuchardt , W. Blankenfeldt , A. K. H. Hirsch , F. Pessler , Nat. Metab. 2022, 4, 534. 35655026 10.1038/s42255-022-00577-x PMC9170585

[83] P. Singh , K. Gollapalli , S. Mangiola , D. Schranner , M. A. Yusuf , M. Chamoli , S. L. Shi , B. Lopes Bastos , T. Nair , A. Riermeier , E. M. Vayndorf , J. Z. Wu , A. Nilakhe , C. Q. Nguyen , M. Muir , M. G. Kiflezghi , A. Foulger , A. Junker , J. Devine , K. Sharan , S. J. Chinta , S. Rajput , A. Rane , P. Baumert , M. Schönfelder , F. Iavarone , G. di Lorenzo , S. Kumari , A. Gupta , R. Sarkar , et al., Science 2023, 380, abn9257. 10.1126/science.abn9257 PMC10630957 37289866

[84] K. E. de Goede , K. J. Harber , F. S. Gorki , S. G. S. Verberk , L. A. Groh , E. D. Keuning , E. A. Struys , M. van Weeghel , A. Haschemi , M. P. J. de Winther , X. A. M. H. van Dierendonck , J. Van den Bossche , Biochim Biophys Acta Mol Basis Dis 2022, 1868, 166427. 35526742 10.1016/j.bbadis.2022.166427

[85] J. H. Wang , M. Doyle , B. J. Manning , Q. Di Wu , S. Blankson , H. P. Redmond , J. Biol. Chem. 2002, 277, 3606875. 10.1074/jbc.M205584200 12133836

[86] Y. Fan , G. Zhang , C. T. Vong , R. D. Ye , Am J Physiol Lung Cell Mol Physiol 2020, 318, L314. 31851532 10.1152/ajplung.00309.2019

[87] M. J. Shin , D. W. Kim , Y. J. Choi , H. J Cha , S. H Lee , S. Lee , J. Park , K. H. Han , W. S. Eum , S. Y. Choi , BMB Rep. 2020, 53, 106. 31964467 10.5483/BMBRep.2020.53.2.180 PMC7061214

[88] J. Jing , I. V. Yang , L. Hui , J. A. Patel , C. M. Evans , R. Prikeris , L. Kobzik , B. P. O'Connor , D. A. Schwartz , J. Immunol. 2013, 190, 63607. 10.4049/jimmunol.1202942 PMC3679202 23667110

[89] E. Weiss , K. Schlatterer , C. Beck , A. Peschel , D. Kretschmer , J Infect Dis 2020, 221, 668. 31573600 10.1093/infdis/jiz498

[90] S. Han , H. Zhuang , S. Shumyak , J. Wu , H. Li , L‐J Yang , W. H. Reeves , J. Immunol. 2017, 199, 12614. 10.4049/jimmunol.1700099 PMC5548631 28696256

[91] I. Akhmetzyanova , M. J. McCarron , S. Parekh , M. Chesi , P. L. Bergsagel , D. R. Fooksman , Leukemia 2020, 34, 245. 31439945 10.1038/s41375-019-0519-4 PMC6923614

[92] X. Liu , W. Xu , J. Feng , Y. Wang , K. Li , Y Chen , W. Wang , W. Zhao , S. Ge , J. Li , Nat. Commun. 2025, 16, 1363. 39905015 10.1038/s41467-025-56460-2 PMC11794888

[93] J. Liu , J. M. Buckley , H. P. Redmond , J. H. Wang , J. Immunol. 2010, 184, 58028. 10.4049/jimmunol.0904127 20400705

[94] A. Butler , P. Hoffman , P. Smibert , E. Papalexi , R. Satija , Nat. Biotechnol. 2018, 36, 411. 29608179 10.1038/nbt.4096 PMC6700744

[95] T. Wu , E. Hu , S. Xu , M. Chen , P. Guo , Z. Dai , T. Feng , L. Zhou , W. Tang , L. Zhan , X. Fu , S. Liu , X. Bo , G. Yu , Innovation (Camb) 2021, 2, 100141. 34557778 10.1016/j.xinn.2021.100141 PMC8454663

[96] A. S. Castanza , J. M. Recla , D. Eby , H. Thorvaldsdottir , C. J. Bult , J. P. Mesirov , Nat. Methods 2023, 20, 16190. 10.1038/s41592-023-02014-7 PMC11397807 37704782

[97] F. Zhang , X. Li , W. Tian , Cell Rep. 2020, 32, 108069. 32846127 10.1016/j.celrep.2020.108069

[98] L. Zeng , R. Kang , S. Zhu , X. Wang , L. Cao , H. Wang , T. R. Billiar , J. Jiang , D. Tang , Sci. Transl. Med. 2017, 9, aan5689. 10.1126/scitranslmed.aan5689 PMC5737927 29046432

[99] S. Wang , D. Jiang , F. Huang , Y. Qian , M. Qi , H. Li , X. Wang , Z. Wang , K. Wang , Y. Wang , P. Du , B. Zhan , R. Zhou , L. Chu , X. Yang , Parasit Vectors 2023, 16, 450. 38066526 10.1186/s13071-023-06021-7 PMC10709918

[100] J. Liu , Y. Wang , L. Zeng , C. Yu , R. Kang , D. J. Klionsky , J. Jiang , D. Tang , Autophagy 2024, 20, 2616. 38916095 10.1080/15548627.2024.2372215 PMC11587848

