# Supplementary material for: Exploring Functionally Enhanced BLP‐Trained Macrophage Subpopulations in S. Aureus Infection: Underlying Mechanisms and Therapeutic Significance
Source: Adv Sci (Weinh). 2025 Oct 21;12(47):e17142. doi: 10.1002/advs.202417142 (PMC12713031; doi:10.1002/advs.202417142)
Supplement: Supplementary file 1 — Supporting Information [file ADVS-12-e17142-s002.pdf]

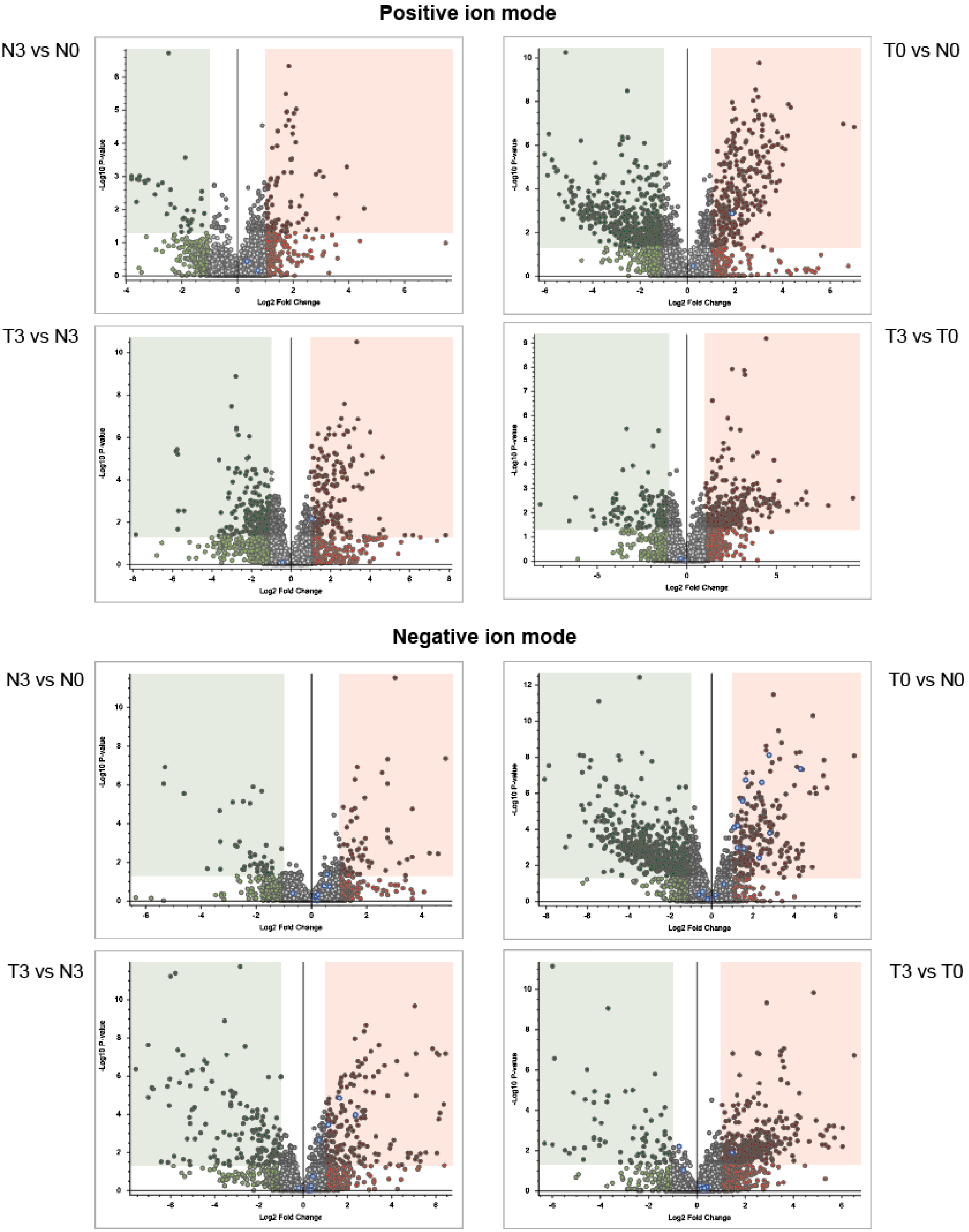

Volcano plot analysis of metabolomic profiles across experimental groups

A Positive ion mode detection. **B** Negative ion mode detection. Each point represents a detected ion, with the x-axis corresponding to log$_2$(fold change) and the y-axis indicating statistical significance (-log$_{10}$[*p*-value]). Red or Green backgroud marks thresholds for significance (|fold change|>2, *p*< 0.05).

RT-PCR Primer Sequences

Primer Name	Base Sequence (5'to3')
MARCO-F	*ACAGAGCCGATTTTGACCAAG*
MARCO-R	*CAGCAGTGCAGTACCTGCC*
SLPI-F	*GGCCTTTTACCTTTCACGGTG*
SLPI-R	*TACGGCATTGTGGCTTCTCAA*
FPR1-F	*CATTTGGTTGGTTCATGTGCAA*
FPR1-R	*AATACAGCGGTCCAGTGCAAT*
CD38-F	*TCTCTAGGAAAGCCCAGATCG*
CD38-R	*GTCCACACCAGGAGTGAGC*
ADGRE1-F	*TGACTCACCTTGTGGTCCTAA*
ADGRE1-R	*CTTCCCAGAATCCAGTCTTTCC*
IFITM2-F	*TGGGCTTCGTTGCCTATGC*
IFITM2-R	*AGAATGGGGTGTTCTTTGTGC*
CTSC-F	*CAACTGCACCTACCCTGATCT*
CTSC-R	*TAAAATGCCCGGAATTGCCCA*
PRDX6-F	*CGCCAGAGTTTGCCAAGAG*
PRDX6-R	*TCCGTGGGTGTTTCACCATTG*
SLC7A11-F	*GGCACCGTCATCGGATCAG*
SLC7A11-R	*CTCCACAGGCAGACCAGAAAA*
SOD2-F	*CAGACCTGCCTTACGACTATGG*
SOD2-R	*CTCGGTGGCGTTGAGATTGTT*
HMOX1-F	*AAGCCGAGAATGCTGAGTTCA*
HMOX1-R	*GCCGTGTAGATATGGTACAAGGA*
CLEC4E-F	*AGTGCTCTCCTGGACGATAG*
CLEC4E-R	*CCTGATGCCTCACTGTAGCAG*
CLEC4A1-F	*GACTCGTCTTCATGTACCGTCT*
CLEC4A1-R	*AGCAACAGAGAATAAGATTGCCA*
PKM2-F	*TTAGGCCAGCAACGCTTGTAGTGC*
PKM2-R	*AGATGCTGCCGCCCTTCTGTGATA*
LDHA-F	*CACTGACTCCTGAGGAAGAGGCCC*
LDHA-R	*AGCTCAGACGAGAAGGGTGTGGTC*
HIF1A-F	*ACCTTCATCGGAAACTCCAAAG*
HIF1A-R	*CTGTTAGGCTGGGAAAAGTTAGG*
SDH-F	*TACAAAGTGCGGGTCGATGA*
SDH-R	*TGTTCCCCAAACGGCTTCT*
IRG1-F	*GCGAACGCTGCCACTCA*
IRG1-R	*ATCCCAGGCTTGGAAGGTC*
18S-F	*AGTCCCTGCCCTTTGTACACA*
18S-R	*CGATCCGAGGGCCTCACTA*
PRDX1-F	*AATGCAAAAATTGGGTATCCTGC*
PRDX1-R	*CGTGGGACACACAAAAGTAAAGT*
GCLM-F	*AGGAGCTTCGGGACTGTATCC*
GCLM-R	*GGGACATGGTGCATTCCAAAA*
CFP-F	*TTCACCCAGTATGAGGAGTCC*
CFP-R	*GCTGACCATTGTGGAGACCT*
CLEC4A2	*CCCCCATTGGACAAAGGGC*
CLEC4A2	*GGTGCCAAGATACCCAAGTCTA*
