# Supplementary material for: Exploring Functionally Enhanced BLP‐Trained Macrophage Subpopulations in S. Aureus Infection: Underlying Mechanisms and Therapeutic Significance
Source: Adv Sci (Weinh). 2025 Oct 21;12(47):e17142. doi: 10.1002/advs.202417142 (PMC12713031; doi:10.1002/advs.202417142)
Supplement: Supplementary file 2 — Supporting Information [file ADVS-12-e17142-s001.docx]

**Exploring Functionally Enhanced BLP-Trained Macrophage Subpopulations in *S. aureus*** **Infection: Underlying Mechanisms and Therapeutic Significance**

Yantong Wan^1,2†^, Yinghao Hong^1†^, Xiangjun Ji^1,2†^, Jing Xiang^1^, Jinxi Liu^1^, Lixin Liang^1^, Meng Ren^3^, Wenhan Chen^1^, Tengfei Xu^4^, Zhijie Li^4^, Tieliu Shi^3^, Yong Jiang^1,5^*, Huaping Liang^2^*, Jinghua Liu^1^*

^1^ Guangdong Provincial Key Laboratory of Proteomics, Department of Pathophysiology, School of Basic Medical Sciences, Southern Medical University, Guangzhou, Guangdong, 510515, China.

^2^ State Key Laboratory of Trauma and Chemical Poisoning, Daping Hospital, Army Medical University, Chongqing, 400038, China.

^3^ Center for Bioinformatics and Computational Biology, The Institute of Biomedical Sciences and School of Life Sciences, East China Normal University, Shanghai, 200062, China.

^4^ Department of Geriatric Medicine, Shenzhen People’s Hospital, the Second Clinical Medical College, Jinan University, Shenzhen, Guangdong, 518020, China.

^5^ Department of Respiratory and Critical Care Medicine, The Tenth Affiliated Hospital, Southern Medical University, Dongguan, Guangdong, 523018, China.

† These authors have contributed equally to this work and share ﬁrst authorship

***Correspondence:** Jinghua Liu at liujhua@smu.edu.cn; Huaping Liang at Lianghuaping@tmmu.edu.cn; or Yong Jiang at yjiang@i.smu.edu.cn.


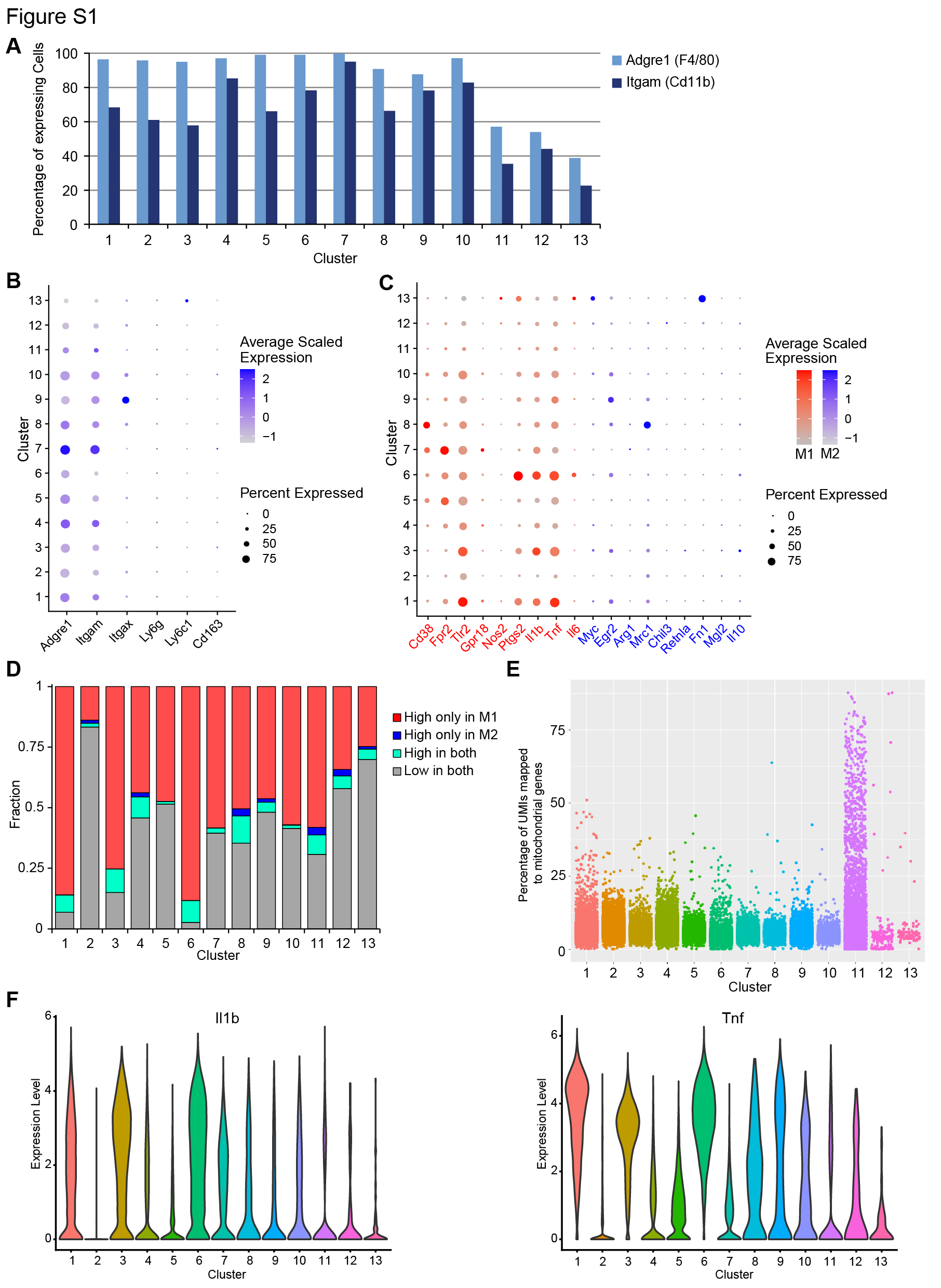


**Supplementary Figure 1. Single-cell transcriptional profiling identifies 13 populations across naive and BLP-trained macrophages. A** Percentage of macrophage markers expression (Adgre1, Itgam) in each cluster. **B** Bubble plot shows normalized expression of Adgre1, Itgam, Itgax, Ly6g, Ly6c1, and Cd163 across clusters at resting state. **C** Bubble plot shows scaled normalized expression of M1 (red) and M2 (blue) markers across clusters. **D** Stacked bar plot with fractions of different types of macrophages (relative to the total number of cells in each cluster) shown in the y-axis. **E** Percentage of UMIs (unique molecular identifier) mapped to mitochondrial genes per cell across different clusters. **F** Distribution of log-transformed gene expression of *Tnf* and *Il1b* in all clusters.

**
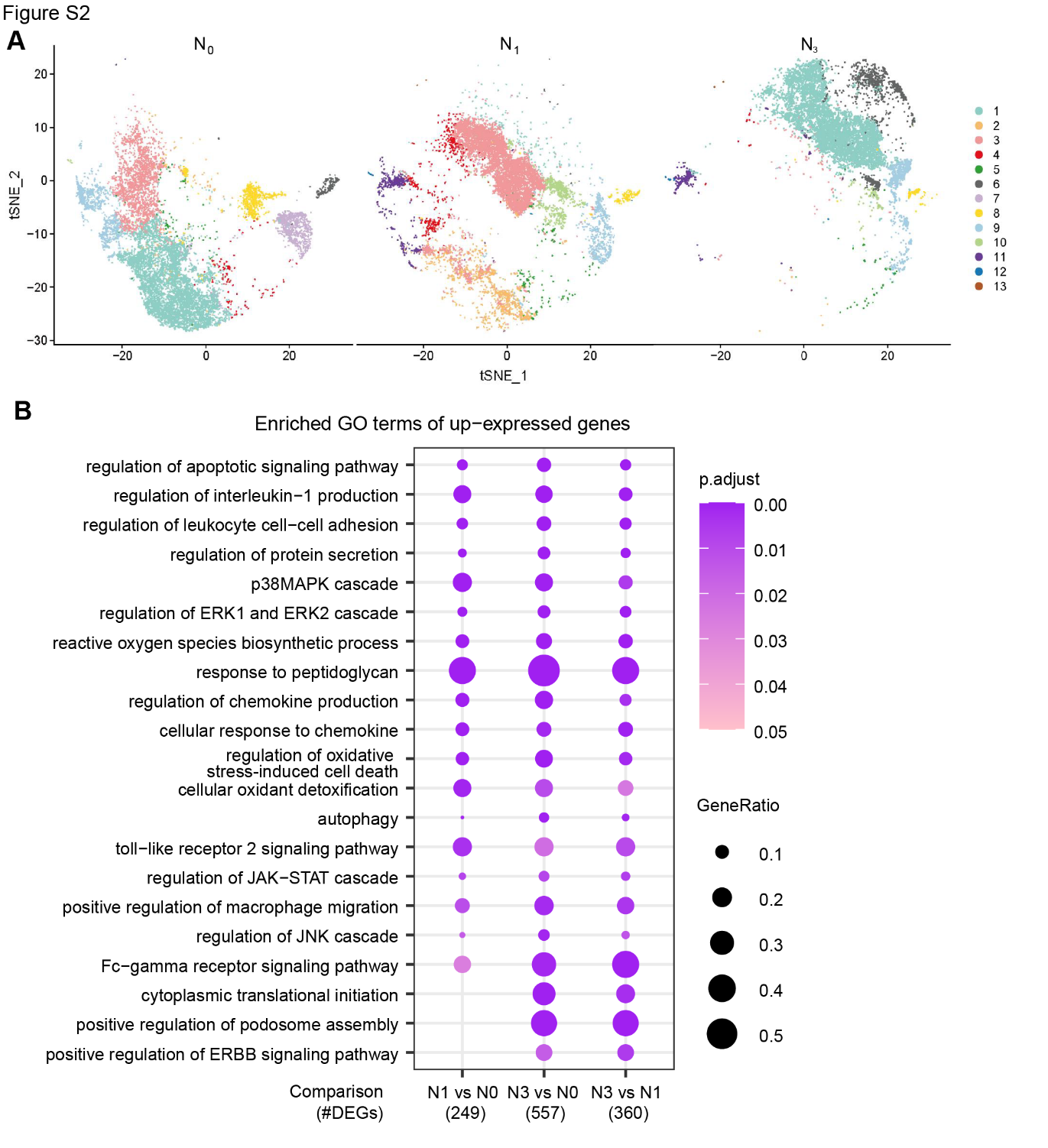
**

**Supplementary Figure 2. Subpopulation characteristics of naïve macrophages in the resting status and after bacterial infection.** **A** Visualization of the distribution of subpopulations at N0, N1, and N3. **B** GO enrichment analysis of highly upregulated genes (lnFC > 0.25) in macrophages infected with *S. aureus* at 1, 3 h when compared with uninfected macrophages. Enriched gene numbers are indicated in brackets. N0, Naïve macrophages without bacterial infection. N1 and N3, Naïve BMDMs treated with bacterial for 1 h and 3 h.


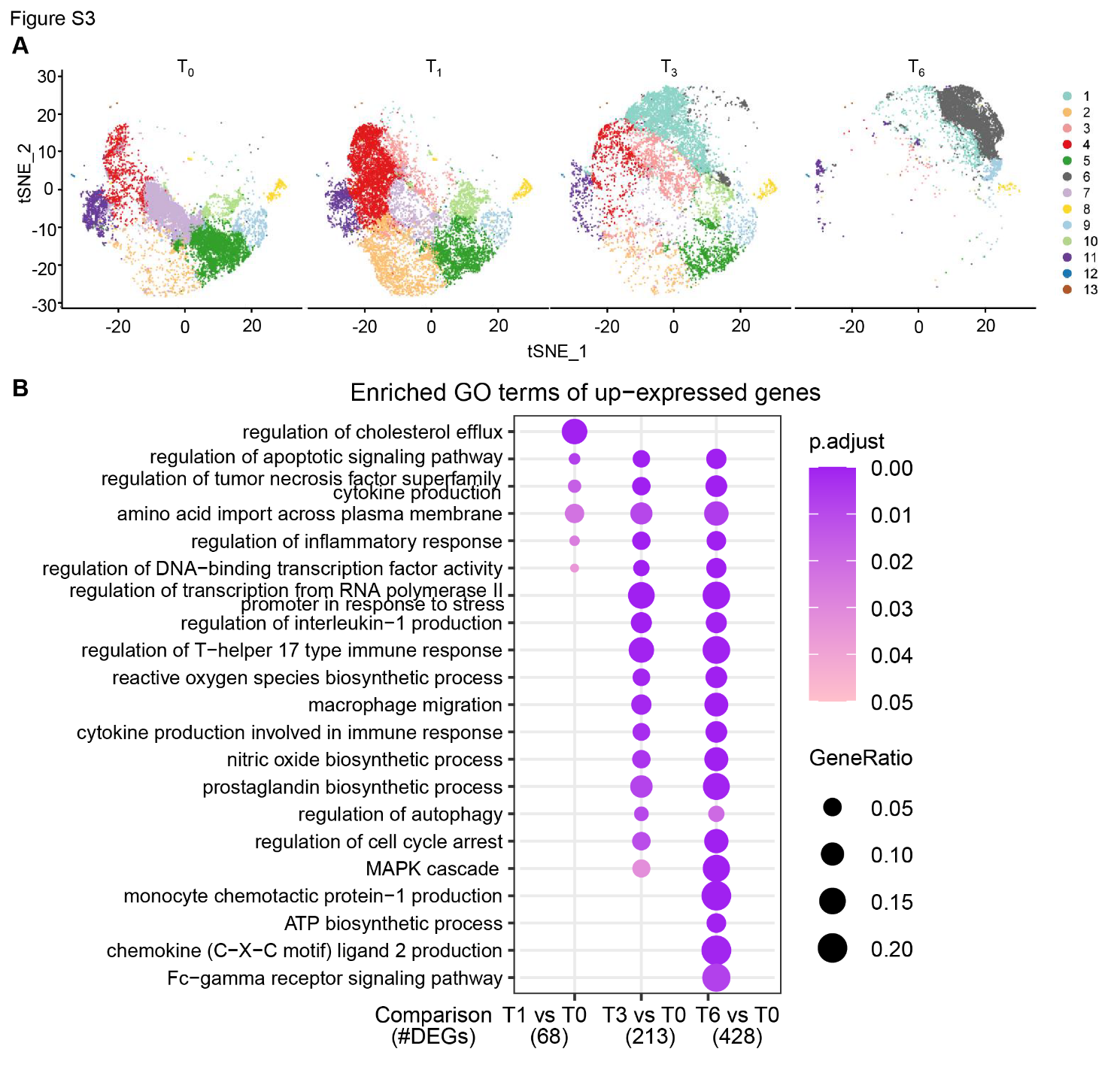


**Supplementary Figure 3. Subpopulation characteristics of BLP-trained macrophages in the resting status and after bacterial infection. A** Visualization of the distribution of BLP-trained subpopulations during *S. aureus* infection. **B** GO enrichment analysis of highly upregulated genes (lnFC > 0.25) in the BLP trained macrophages infected with *S. aureus* at 1, 3, 6 h compared with uninfected BLP trained macrophages. Enriched gene numbers are indicated in brackets.

**
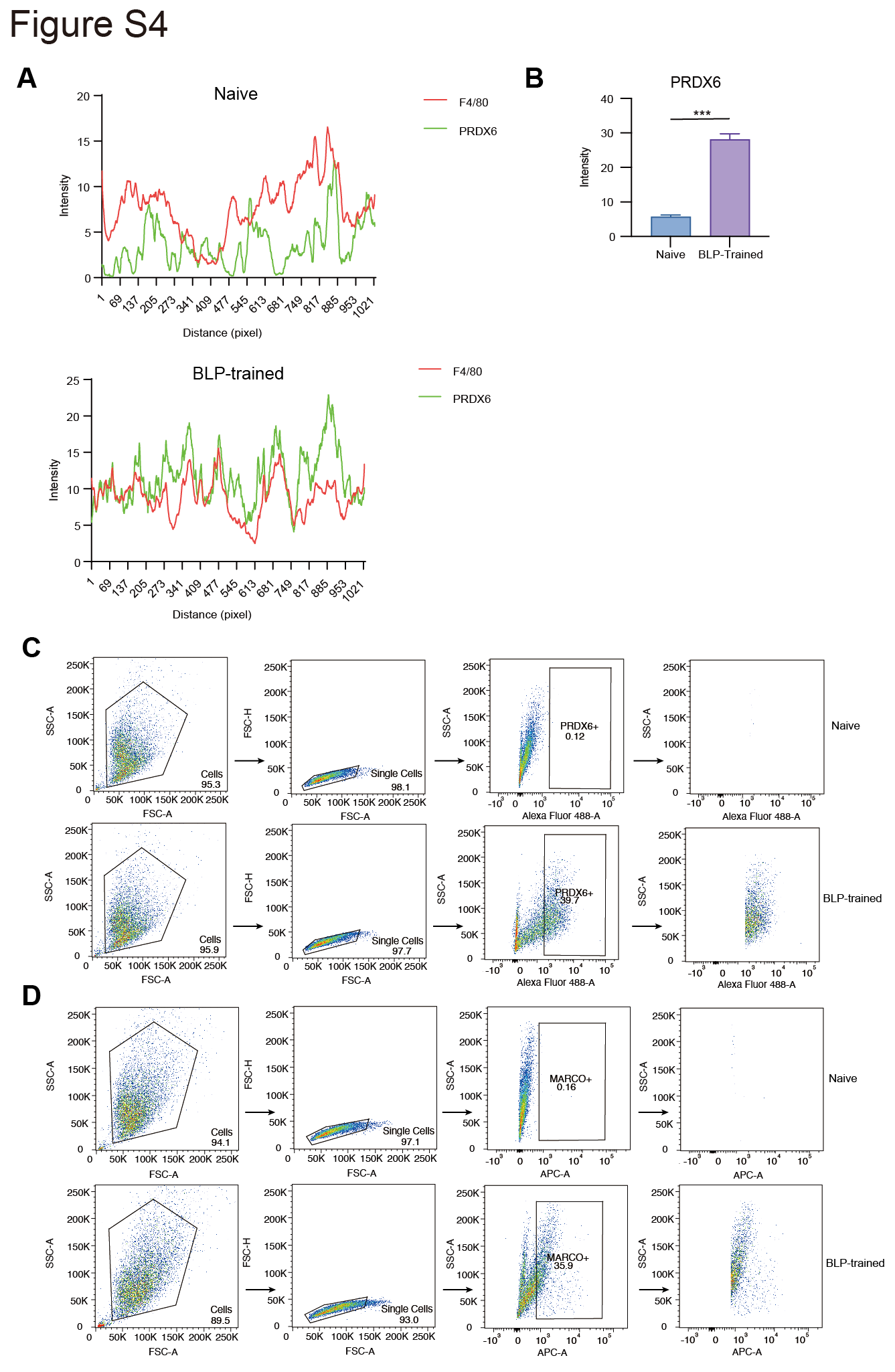
**

**Supplementary Figure 4. Two specific subpopulations induced by BLP-training and their characteristics. A** Immunofluorescence analysis of PRDX6 expression and its colocalization with F4/80 in naive versus BLP-trained BMDMs. **B** Quantitative immunofluorescence assessment of PRDX6 protein levels in naive and BLP-trained BMDMs. **C, D** Flow cytometric gating strategy for PRDX6-positive cells and MARCO-positive cells. Representative dot plots show the gating strategy used to identify PRDX6^high^ cells (C) and MARCO^high^ cells (D). The initial gating was performed on viable cells based on the forward scatter (FSC) and side scatter (SSC), followed by exclusion of debris and doublets using FSC-A vs FSC-H. PRDX6^high^ and MARCO^high^ cells were identified by their high fluorescence intensity, with the gate setting to capture the top 30% of the population, as indicated by the dotted line.

**
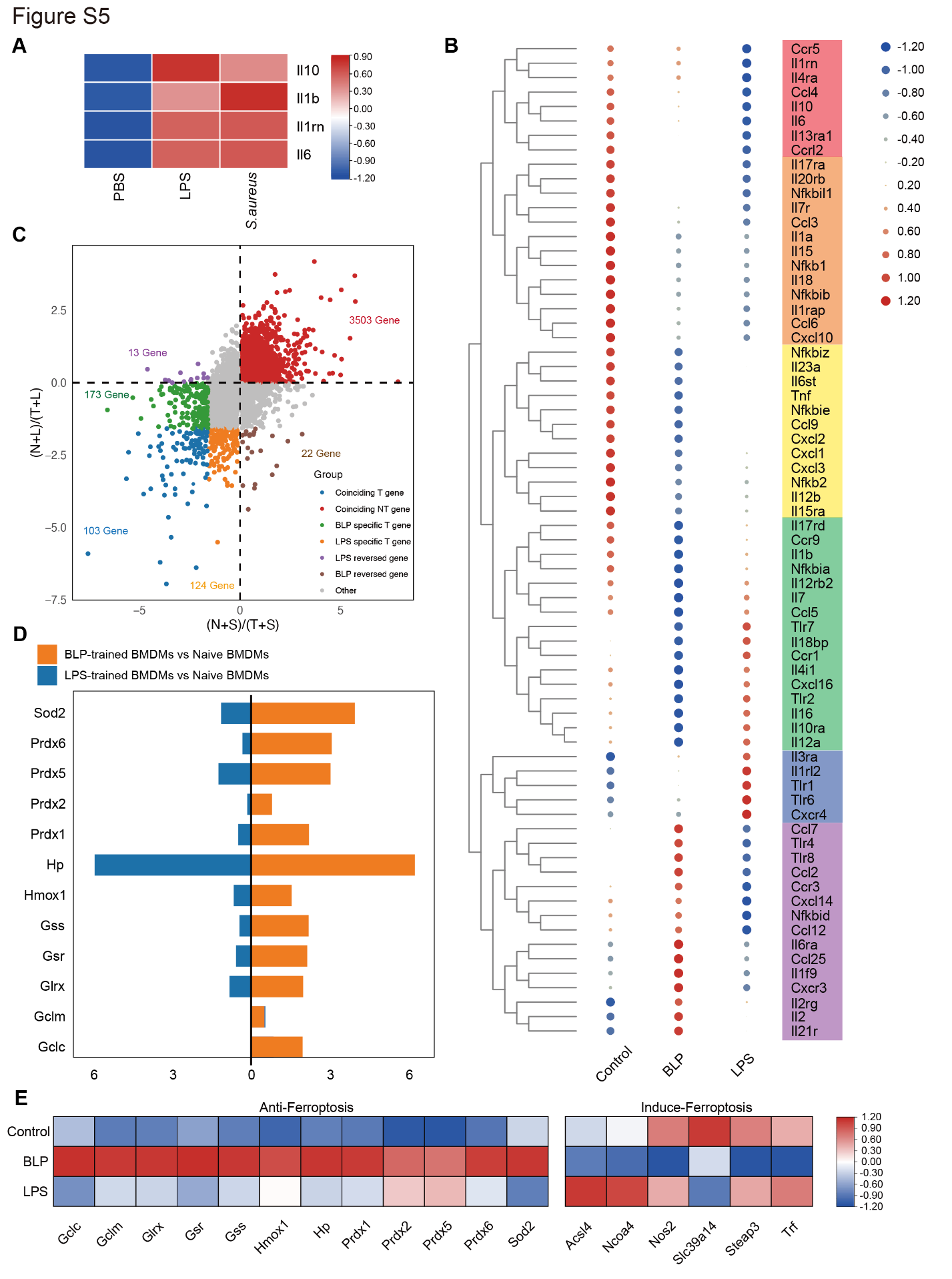
**

**Supplementary Figure 5.** **Comparative Analysis of BLP and LPS Training in Macrophages.** **A** Heatmap illustrating the mRNA transcription levels of inflammatory cytokines *Il10*, *Il1b*, *Il1rn*, and *Il6* following 3 h treatment with LPS or *S. aureu*s infection. Values for the control group were set to zero, and data were standardised by row. **B** Heatmap depicting clustering of inflammation-related genes during secondary challenge in BLP-trained macrophages and LPS-trained macrophages. “BLP” denotes the log₂ fold change (LogFC) between BLP-trained macrophages and naïve macrophages, both infected with S. aureus for 3 h. “LPS” denotes the LogFC between LPS-trained macrophages and naïve macrophages, both treated with LPS for 3 h. Control values were set to zero, and data were standardised by row. **C** Scatter plot comparing gene expression changes in BLP-trained macrophages (x-axis: LogFC versus naïve macrophages following *S. aureus* infection for 3 h) and in LPS-trained macrophages (y-axis: LogFC versus naïve macrophages following 3 h LPS stimulation). Genes were categorised as T (tolerisable) or NT (non-tolerisable) based on initial definitions: T genes were defined as (naïve + stimulus)/(tolerant + stimulus) > 3; NT genes as (naïve + stimulus)/(tolerant + stimulus) < 1. **D** Bar graph comparing expression of oxidative stress-associated proteins at resting state between BLP-trained macrophages and LPS-trained macrophages. Orange bars represent LogFC values for BLP-trained versus naïve macrophages; blue bars represent LogFC values for LPS-trained versus naïve macrophages.  **E** Heatmap showing ferroptosis-resistance capacity during secondary challenge in BLP-trained macrophages and LPS-trained macrophages. Definitions for ‘BLP’ and ‘LPS’ are as described in panel B. Control values were set to zero, and data were standardised by column.

**
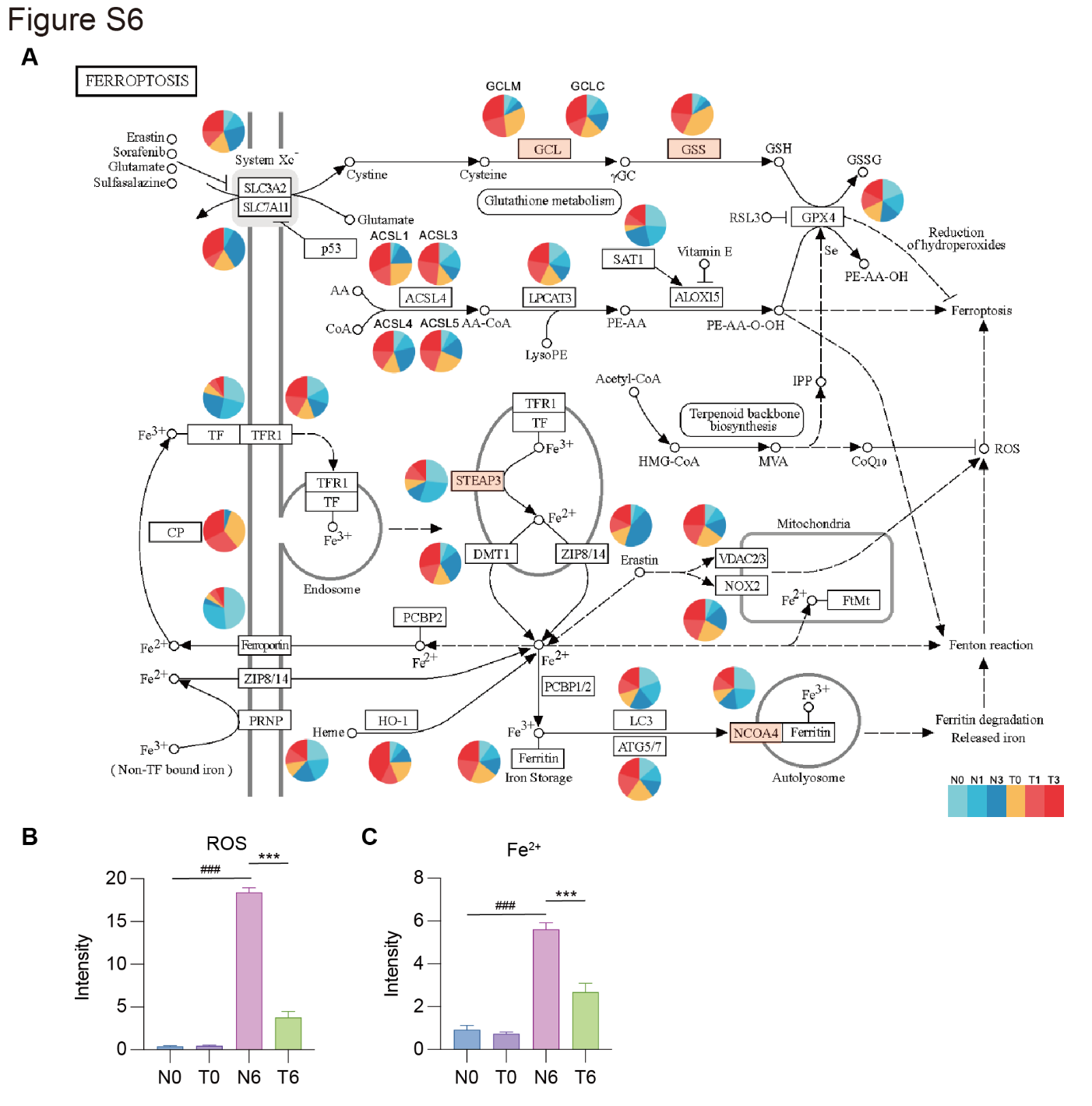
**

**Supplementary Figure 6. BLP-trained tolerance attenuates *S.aureus* infection-initiated ferroptosis. A** Mapping ferroptosis pathways using single-cell RNA-seq and KEGG mapper. **B, C** Immunofluorescence intensity analysis of ROS and Fe^2+^.

**
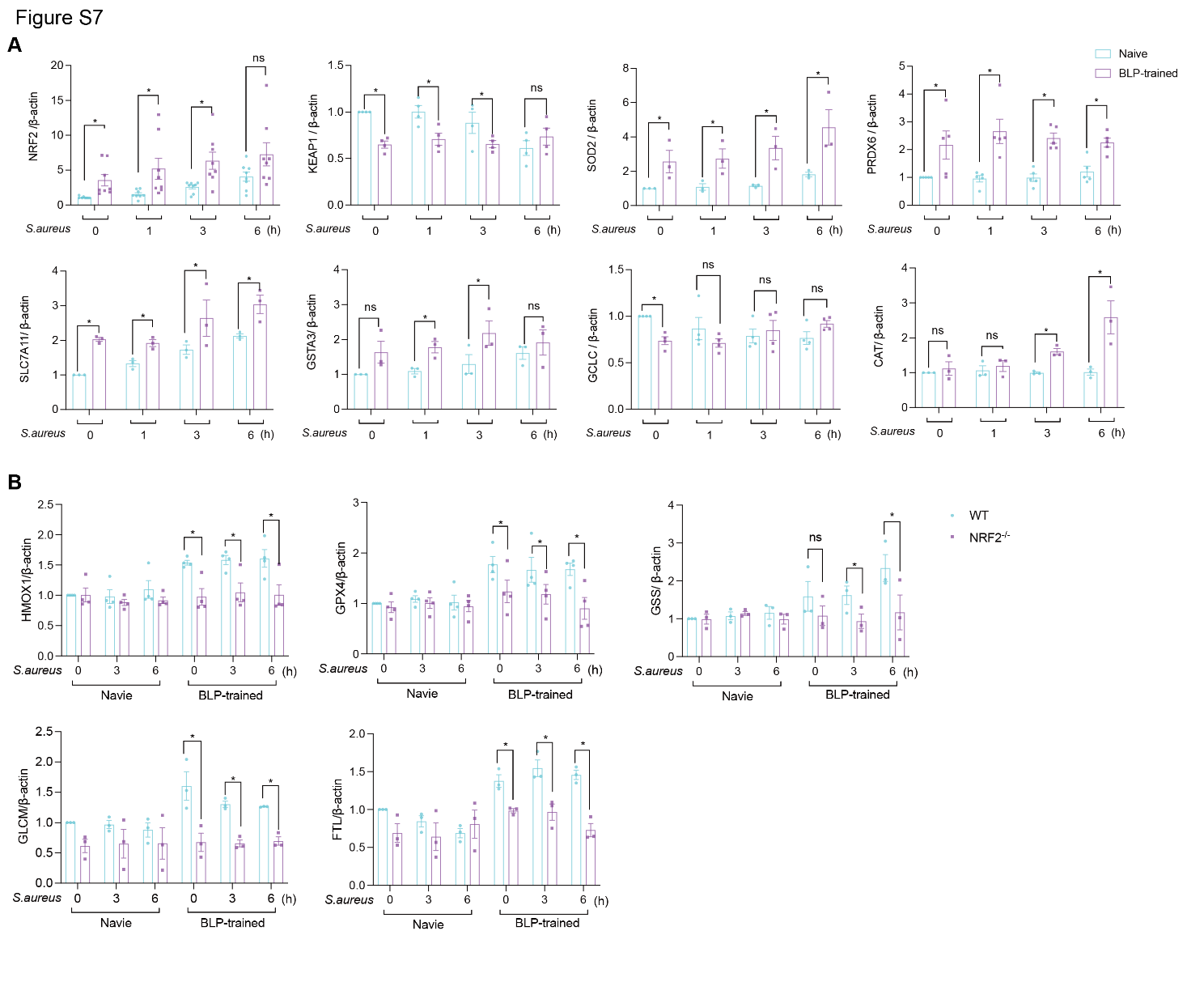
**

**Supplementary Figure 7. Quantification of Western blot analysis in Figure 7F and 7H. A, B** Quantification of Nrf2, KEAP1, SOD2, PRDX6, SLC7A11, GSTA3, GCLC, and CAT (from Figure 7F) (A) andNrf2, HMOX1, GPX4, GSS, GCLM, and FTL (from Figure 7H) (B). Quantification was performed by densitometric analysis, with target protein band intensities normalized to β-actin and expressed as relative fold change compared to naive BMDMs. Data are presented as mean ± SEM (n ≥ 3), with **p* < 0.05 determined by the nonparametric Mann-Whitney test.

**
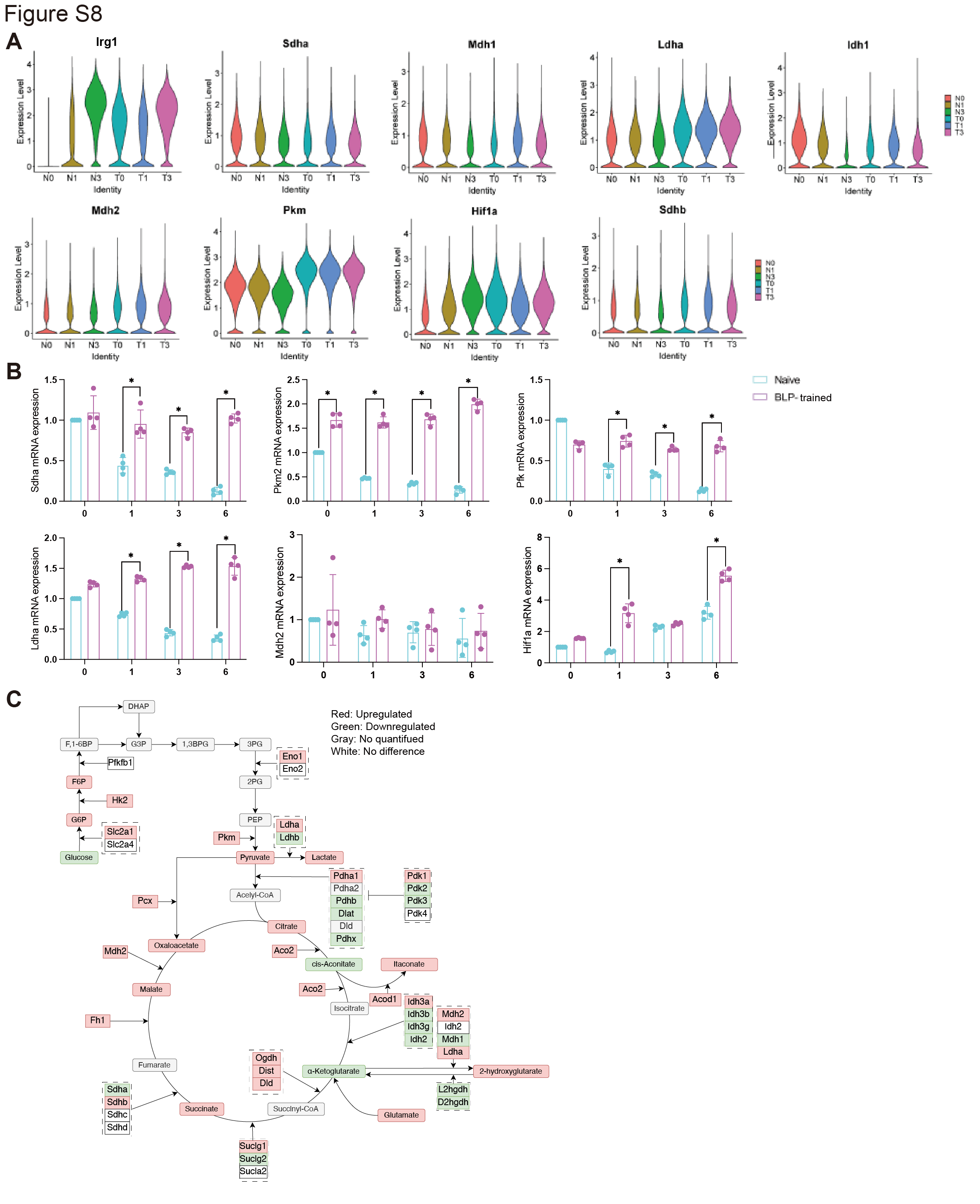
**

**Supplementary Figure 8.** **BLP-trained macrophages undergo metabolic reprogramming. A** The violin diagram shows the changes in key metabolic enzymes in the groups N0, N1, N3, T0, T1, and T3. **B** mRNA levels of the metabolism-related enzymes in BLP-trained macrophages and naive macrophages infected with *S. aureus* for 0, 1, 3, 6 h using RT-qPCR experiments. **C** The gene expression level in the figure is obtained from the single-cell transcriptome sequencing analysis of BLP training-tolerant BMDMs and compared with the N group of cells; the metabolite abundance is obtained from the metabolomics analysis and the comparison of the two groups. In the figure, red represents the genes and metabolites up-regulated by BLP training compared with the control; green represents down-regulation; no significant difference is white; and unquantified genes and metabolites are shown in gray. The results shown represent one experiment from a total of three separate experiments. All data are presented as the mean ± SD of three separate experiments. **p*< 0.05 compared to naive BMDMs at the same time point as bacterial infection.

**
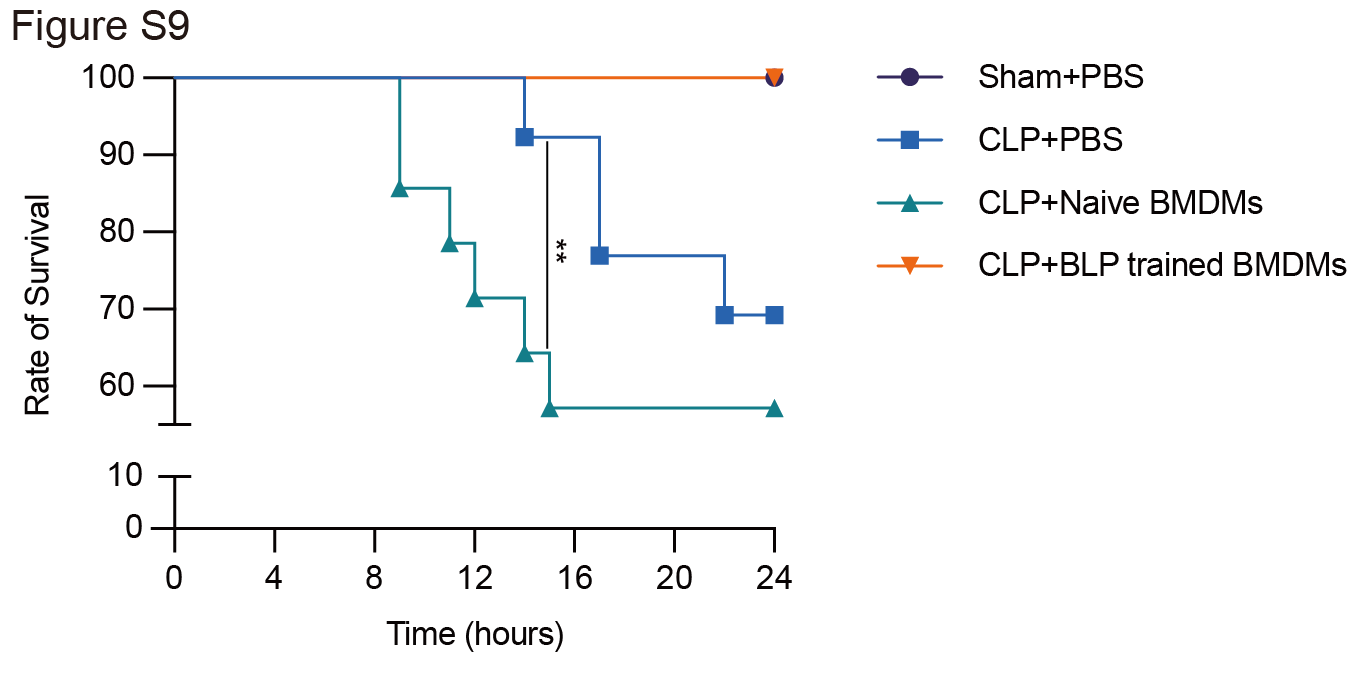
**

**Supplementary Figure 9. Early survival rate in septic mice following transfer of Naive Macrophages.** Compared with PBS-treated mice (n=13), mice adoptively transferred naive BMDMs (n=14) showed significantly higher mortality at 15 h after CLP-induced severe microbial sepsis (*p*=0.01).

**Supplementary Table 1. Number of cells in different clusters.**

**Supplementary Table 2. Differentially expressed genes (DEGs) of each Cluster and their GO enrichment analysis.**

**Supplementary Table 3. Differentially-expressed genes in each N group.**

**Supplementary Table 4. Differentially-expressed genes in each T group.**

**Supplementary Table 5. Signature genes (*p*_val_adj < 0.05, avg_log2FC > 0) of C5 and C7.**

**Supplementary Table 6. Differentially expressed genes between C5 and C7.**

**Supplementary Table 7. Identification of tolerizeable and non-tolerizeable genes in BLP-trained BMDMs.**

**Supplementary Table 8. Differences in gene expression between BLP training and LPS training with secondary stimulation.**

**Supplementary file. Volcano plot analysis of metabolomic profiles across experimental groups and primer sequences for RT-qPCR**
